# Sir2 Paralogues Cooperate to Regulate Virulence Genes and Antigenic Variation in *Plasmodium falciparum*

**DOI:** 10.1371/journal.pbio.1000084

**Published:** 2009-04-14

**Authors:** Christopher J Tonkin, Céline K Carret, Manoj T Duraisingh, Till S Voss, Stuart A Ralph, Mirja Hommel, Michael F Duffy, Liliana Mancio da Silva, Artur Scherf, Alasdair Ivens, Terence P Speed, James G Beeson, Alan F Cowman

**Affiliations:** 1 The Walter and Eliza Hall Institute of Medical Research, Melbourne, Australia; 2 The Wellcome Trust Sanger Institute, Cambridge, United Kingdom; 3 Department of Biochemistry, Bio21 Institute, The University of Melbourne, Melbourne, Australia; 4 Department of Medicine RMH/WH, The University of Melbourne, Melbourne, Australia; 5 Unité de Biologie des Interactions Hôte-Parasite, Institut Pasteur and CNRS, Paris, France; Cornell University, United States of America

## Abstract

Cytoadherance of Plasmodium falciparum-infected erythrocytes in the brain, organs and peripheral microvasculature is linked to morbidity and mortality associated with severe malaria. Parasite-derived P. falciparum Erythrocyte Membrane Protein 1 (PfEMP1) molecules displayed on the erythrocyte surface are responsible for cytoadherance and undergo antigenic variation in the course of an infection. Antigenic variation of PfEMP1 is achieved by *in situ* switching and mutually exclusive transcription of the *var* gene family, a process that is controlled by epigenetic mechanisms. Here we report characterisation of the P. falciparum silent information regulator's A and B (PfSir2A and PfSir2B) and their involvement in mutual exclusion and silencing of the *var* gene repertoire. Analysis of P. falciparum parasites lacking either PfSir2A or PfSir2B shows that these NAD^+^-dependent histone deacetylases are required for silencing of different *var* gene subsets classified by their conserved promoter type. We also demonstrate that in the absence of either of these molecules mutually exclusive expression of *var* genes breaks down. We show that *var* gene silencing originates within the promoter and PfSir2 paralogues are involved in *cis* spreading of silenced chromatin into adjacent regions. Furthermore, parasites lacking PfSir2A but not PfSir2B have considerably longer telomeric repeats, demonstrating a role for this molecule in telomeric end protection. This work highlights the pivotal but distinct role for both PfSir2 paralogues in epigenetic silencing of P. falciparum virulence genes and the control of pathogenicity of malaria infection.

## Author Summary

The unicellular parasite Plasmodium falciparum is the cause of the most severe form of malaria and is responsible for 300 million infections and ∼2 million deaths a year. Infected erythrocytes clump and block capillaries in the peripheral circulation, the brain, and placenta and are a major contributor to the pathology of malaria. A parasite-derived protein displayed on the surface of the infected erythrocyte is responsible for erythrocyte clumping in capillaries. Although 60 subtelomeric *var* genes can encode different versions of this “sticky” capillary-binding protein, only one protein is expressed at a time, and switches in expression between these genes causes variation of this pathogenic molecule enabling the parasite to evade the immune system. Here we identify two chromatin-modifying proteins that cooperate to mediate silencing and mutual exclusive expression of *var* genes. These proteins are thus important virulence factors of the malaria-causing parasite.

## Abstract 3

Investigation into two Sir2 histone deacetylases in the malaria-causing parasite reveals*trans*-acting epigenetic factors control mutually exclusive expression of a major subtelomeric virulence gene family.

## Introduction

Phenotypic variation is essential for survival in a changing and competitive environment. In the case of pathogenic organisms, antigenic variation of cell surface proteins is a common mechanism to avoid clearance by the host immune system (for review see [1]). Although many different strategies exist for the regulation of antigenic variation, most rely on the mutually exclusive expression of gene families, that is, the expression of one variant and the silencing of all others [1,2]. By switching antigenic variants pathogens are able to avoid adaptive immune responses, thus maintaining a persistent infection and increasing the chances of transmission to the next host.

The protozoan parasite P. falciparum, the causative agent of the most severe form of malaria in humans, undergoes antigenic variation in its asexual blood stage, an important property of the parasite both for virulence and evasion of host immune responses (for review see, [3]) [4]. P. falciparum infects up to 300 million people a year resulting in the deaths of over 2 million annually [5]. Infection can manifest in a variety of ways and in severe cases cerebral abnormalities and organ failure can occur, which often leads to death. Such complications of infection are multifactorial but cytoadherance of infected red blood cells to the microvasculature and to uninfected erythrocytes plays an important role [3]. Parasite-derived P. falciparum Erythrocyte Membrane Protein 1 (PfEMP1) is exposed on the surface of the infected red blood cell and responsible for cytoadherance to receptors on the microvasculature, brain, and placenta. Capillary blockages and local inflammatory responses ensue and disease progresses. Thus, PfEMP1 is a key P. falciparum virulence factor and an important target of the host immune system [4].

To maintain cytoadherance in the presence of a mounting immune response PfEMP1 undergoes antigenic variation by switching expression between the ∼60-member *var* gene family that encodes these proteins [6–10]. *Var* gene expression is controlled at the level of transcription [11,12] and switching to other genes involves no DNA rearrangements [13]. Like many other antigenically variant gene families in pathogenic organisms, most *var* genes occupy subtelomeric regions of P. falciparum chromosomes where gene silencing and recombination occur at high frequency [14–16]. There are also clusters of *var* gene at more central regions on five of the 14 chromosomes [10,14].

*Var* genes have a highly conserved promoter region that fall into three distinct families on the basis of their physical position and orientation along the chromosome [10,17]. The UpsB promoters control *var* genes that are most telomere proximal and are transcribed towards the centromeres. UpsA promoters and the related but distinct UpsE promoter of the highly conserved placental PfEMP1 variant *var2CSA* drive expression of *var* genes that are also within subtelomeric regions but are transcribed towards the telomere. The UpsC promoter-type controls the expression of internal chromosome *var* genes [17,18]. Prediction of binding phenotypes based on ligand domain structure suggests that PfEMP1 molecules encoded by UpsB and UpsC *var* genes most commonly bind the endothelial CD36 and ICAM-1 receptors, whereas UpsA and the related UpsE *var* genes encode PfEMP1 molecules that are thought to bind to other receptors such as complement receptor on red blood cells (causing red blood cell rosetting) and chondroitin sulphate A (CSA) in the case of placental malaria [19,20].

Chromosome ends in P. falciparum are tethered to the nuclear periphery and are clustered into 3–7 distinct foci [15,16]. The subtelomeric DNA repeat rep20 participates in chromosome tethering [21,22] and an unknown proteinaceous molecule(s) binds telomere ends together [16]. Most of the nuclear periphery contains electron dense material consistent with heterochromatin, suggesting that this region is transcriptionally silent [11,23]. Indeed, *var* gene silencing involves heterochromatin formation, and activation of *var* loci correlates with a “relaxation” of nucleosomal architecture and intranuclear repositioning of the activated gene [11,24,25].

Recently, it has been shown that the highly conserved *var* gene promoters are key to mutual exclusive expression of *var* genes [26,27]. There is an indication that the single intron in the conserved genetic structure of *var* genes plays a role in silencing [28], although this is under debate [29]. Certain DNA elements within the highly conserved *var* gene promoters have been shown to bind specific, but yet unidentified proteins that could be involved in *var* activation and silencing [30]. One of these DNA elements has a role in nucleosomal organization although much work remains to decipher the role of *cis*-elements in control of *var* gene expression [27].

Reversible histone-tail modifications and ATP-dependent nucleosome remodelling are important for epigenetic control in eukaryotic gene regulation. Indeed, activation and silencing of *var* genes correlates with specific histone tail marks, suggesting epigenetic memory is involved in maintenance and switching of *var* gene repertoire. Histone H3 lysine 9 acetylation and methylation of lysine 4 have been shown to be associated with *var* gene activation [31], whereas tri-methylation of lysine 9 of histone H3 is associated with *var* gene silencing [31,32].

Given the association of histone modifications with *var* gene expression, molecules that catalyse their addition and removal are likely to be important factors controlling the expression of PfEMP1. The only molecule known to be involved in the regulation of *var* gene transcription is a homologue of the yeast silent information regulator 2 (Sir2) family [23,24]. Sir2 was originally isolated as a suppressor of subtelomeric gene expression in yeast [33] and has subsequently been shown to be a NAD^+^-dependent histone deacetylase [34]. Sir2 molecules, or sirtuins as they are commonly known, are found throughout the tree of life and catalyse the removal of acetyl groups from a variety of substrates in many cellular processes [35]. Enzymatically, PfSir2 represents a classic NAD^+^-dependent histone deacetylase, catalysing the removal of acetyl-groups from histone tails [36]. Some versions also harbour the ability to ribosylate [37], including the previously characterised PfSir2 (here called PfSir2A) [38]. PfSir2 has also been shown to associate with silenced *var* genes and localise to chromosome-end clusters all along the noncoding telomere-associated repeat elements (TARE 1–6) and telomere repeats within the nucleus [23,39]. Furthermore, the genetic ablation of *PfSir2A* in P. falciparum results in the derepression of a subset of *var* genes, thus further implicating histone hyper-acetylation in *var* gene activation and demonstrating PfSir2A as an important virulence factor of P. falciparum infection [23,24].

Very few *trans*-acting factors have been identified that control antigenic variation in pathogenic protozoa, and in this work we characterise the first two that are involved in regulation of *var* gene expression in P. falciparum. We show that two paralogues of the Sir2 NAD^+^-dependent histone deacetylase family cooperate to regulate the expression of the *var* gene repertoire. We show that *var* transcription is regulated by Sir2 paralogues interacting from within conserved promoter regions. Furthermore, we show that Sir2 paralogues also play a part in the regulation of other antigenically variant genes as well as telomere repeat length. Our work highlights these epigenetic factors as key regulators of antigenic variation and thus pathogenicity of the malaria parasite and further provides impetus that other pathogenic protozoa that undergo antigenic variation may also have a similar reliance on such factors for control of their virulence genes.

## Results

### Identification of Sir2 Homologues in P. falciparum

**Figure 1:**
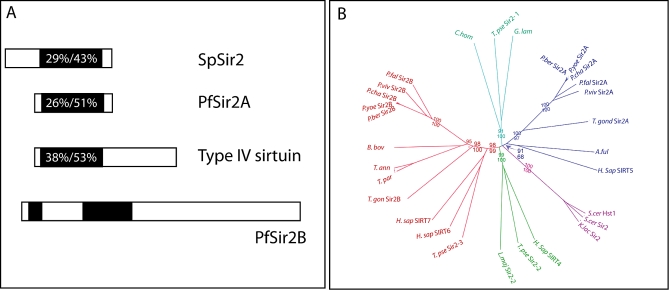
Identification of PfSir2B and Phylogenetic Relationships between PfSir2A and PfSir2B within Apicomplexan Parasites (A) *PfSir2B* is a large gene and encodes a ∼150-kD protein. PfSir2B, like all sirtuins, contains similarity to other Sir2 molecules within the catalytic regions (black). PfSir2B has 29%/43% identity/similarity with Schizosaccharomyces pombe Sir2 (SpSir2) and 26%/51% identity/similarity with the previously identified PfSir2A. PfSir2B most closely resembles a “type IV” sirtuin, which groups together other sirtuins with diverse roles in cellular signalling such as human SIRT6, involved in telomeric chromatin maintenance [82,83] as well as SIRT7, an activator of RNA polymerase I [84]. PfSir2B has with 38%/53% identity/similarity to type IV sirtuins. (B) Phylogenetic analysis of apicomplexan Sir2 paralogues. Sir2 homologues were identified from the sequencing projects of four *Plasmodium* species; P. vivax [85]*, P. chabaudi* [86], *P. yoelli* [86]*, P. berghei* [86], together with Sir2 homologues from other apicomplexan parasites: T. gondii (http://toxodb.org), T. annulata [87]*, T. parva* [87]*, B. bovis* (www.tigr.org), and Cryptosporidium hominis [88]*.* Within the analysis we also included Sir2 from yeast, other related species, and unicellular protozoan parasites. Bootstraps values are indicated for important nodes, where in all cases the upper number is the bootstrap using distance, the lower is the bootstrap using maximum likelihood. “-“ sign indicates a value below 50. Two Sir2 paralogues are a common feature of apicomplexan parasites. Sir2B paralogues (red branches) show strong support for the Sir2B clade, especially within *Plasmodium* species. Sir2B paralogues also groups together type IV sirtuins (SIRT6 and 7) with good support. Sir2A clade (blue branches) groups together Sir2A paralogues of apicomplexan species with excellent support. Sir2A's moderate grouping with SIRT5 and Archaeoglobus fulgidus Sir2-Af1 also shows a possible functional relationship with type III sirtuins. For more in depth analysis see Text S1. P. ber, P. berghei; P. yoe, P. yoellii; P. cha, P. chabaudi; P. viv, P. vivax; P. fal, P. falciparum; T. ann, T. annulata; T. parv, T. parva; T. gondi, T. gondii; B. bov, B. bovis; A. ful, A. fulgidus; H. sap, Homo sapiens; C. hom, C. hominis; T. pse, Thalassiosira pseudonana (diatom); G. lam, Giardia lamblia; K. lac, Kluyveromyces lactis; S. cer, S. cerevisiae; L. maj, Leishmania major.

Previously, it has been shown that a P. falciparum homologue of the histone deacetylase Sir2 (here after referred to as PfSir2A) is involved in the regulation of antigenic variation [23,24]*.* We set out to identify other factors that could be involved in epigenetic regulation of *P. falciparum var* genes. BLAST searching on PlasmoDB (www.plasmodb.org) identified a protein with similarity to PfSir2A and other sirtuins (Figure 1A). This Sir2 homologue, here after referred to as PfSir2B (PlasmoDB accession number PF14_0489), is much larger than PfSir2A and is found on Chromosome 14 (Figure 1A). We compared PfSir2B with the ∼250-amino acid (AA) catalytic domains of sirtuin family members and with PfSir2A. PfSir2B shares 29%/43% identity/similarity with yeast Sir2, a “group I” sirtuin and 26%/51% identity/similarity with PfSir2A, which has previously been defined as a “type III” sirtuin (Figure 1A) [40]. PfSir2B shows the greatest similarity with group IV sirtuins with 38% and 53% identity and similarity, respectively (Figure 1A). Interestingly, the predicted catalytic domain of PfSir2B is split in two, separated by an insertion of approximately 180 amino acids (Figures 1A and Figure S1).

Phylogenetic analysis was performed on Sir2 homologues identified from a range of apicomplexan parasites as well as a diverse selection of eukaryotes. Unrooted trees suggest there are two ancient and distinct apicomplexan Sir2 types. One of these groups strongly with other Sir2 proteins previously described as type IV sirtuins (here Sir2B), while the other does weakly with type III sirtuins (here Sir2A) (Figure 1B). Every *Plasmodium* species analysed, plus Toxoplasma gondi (a coccidian apicomplexan parasite), possesses one of each of these types, while the piroplasm apicomplexans *Theileria* and *Babesia* appear to have secondarily lost their Sir2A paralogue.

What is evident from our analysis is that Sir2 paralogues have been co-opted for the regulation of *var* genes. T. gondii also contains Sir2A and Sir2B orthologues although they are unlikely to be involved in antigenic variation given that no subtelomeric gene family has been identified, even with the genome project near completion (www.toxodb.org). Other *Plasmodium* species analysed here lack *var* genes, but genome projects have revealed other subtelomeric gene families in these parasites that appear to be antigenically variant. We suggest in this work that Sir2 paralogues in P. falciparum directly regulate *var* gene promotors, thus it seems likely that specific factors have co-opted Sir2 paralogues to regulate *var* genes.

To unravel potential similarities and differences in function between Sir2 paralogues an alignment of the catalytic domain was created (Figure S1). We noted that PfSir2B largely has good conservation of residues required for catalytic activity and zinc binding. Residues that represent the acetylated substrate-binding site show some diversity suggesting a change in substrate (Figure S1). For further analysis of the phylogenetic tree and similarities and differences within the catalytic domain between other sirtuins see Text S1. Our analysis clearly demonstrates two Sir2 paralogues are a common feature of malaria parasites and points to a possible role of these molecules in subtelomeric silencing and antigenic variation.

### Disruption of the *PfSir2B* Gene in P. falciparum

**Figure 2:**
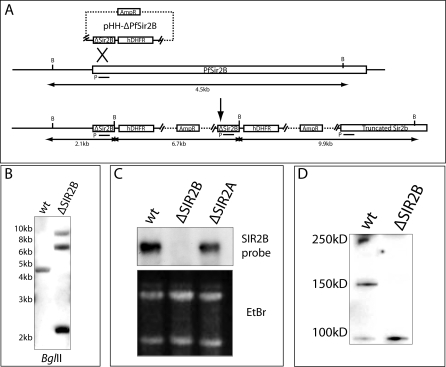
PfSir2B Gene Disruption in P. falciparum Single crossover recombination was used to disrupt *PfSir2B* gene in P. falciparum. Bacterially derived plasmids were introduced containing an ∼1-kb fragment of the 5′ end of the *PfSir2B* gene and an hDHFR expression cassette for stable selection of transgenic parasites. Cycles of “on” and “off” drug were used to select for parasites that had integrated the plasmid by homologous integration into the *PfSir2B* locus. (A) Theoretical restriction mapping of the Sir2B locus, pre- and postepisome integration. Hatched line denotes bacterially derived plasmid sequence, while AmpR signifies beta lactamase expression cassette for selection for resistance on Ampicillin. The line under the 5′ end of the *PfSir2B* gene shows the probe (P) used for Southern blot mapping. B, BglII site. (B) Southern blot of wildtype (wt) and ΔPfSir2B disrupted parasites. Banding pattern is consistent with disruption of the *Sir2B* gene by multiple plasmid copies (as mapped in [A]). DNA ladder sizes are indicated on the left of the blot. (C) Northern blot showing a loss of *PfSir2B* transcript in ΔPfSir2B-disrupted parasites but not in ΔPfSir2A-disrupted parasites. Same probe (P) in Southern blot was used for northern blot. (D) Western blot of antisera raised against a C-terminal portion of PfSir2B. Antisera reacting with a protein in wildtype parasites contain the predicted size for PFSir2B, while this protein is absent as expected in ΔPfSir2B parasites. Cross-reactive bands indicate equal loading of lanes.

To determine the function of PfSir2B in P. falciparum we constructed a parasite line, from the parent strain 3D7, in which the gene encoding the corresponding protein had been disrupted using the plasmid vector pHHΔ*pfsir2B* (Figure 2A) [41]. Southern blot analysis confirmed that the locus had been disrupted by insertion of the transfected plasmid by single crossover homologous recombination (Figure 2B). Furthermore, pulse-field gel electrophoresis showed the plasmid was integrated into a high molecular weight chromosome consistent with Chromosome 14, which encodes *pfsir2B* (Figure S2) [10]. To confirm that the gene had been disrupted we probed RNA from ΔPfSir2B with *pfsir2B* and no specific transcript could be detected, whereas ΔPfSir2A parasites, which encoded an intact *pfsir2A* gene, transcribed similar levels when compared to the parental line 3D7, suggesting there is no compensatory mechanism of Sir2 paralogues (Figure 2C). An antibody derived from the C-terminal portion of PfSir2B recognised a protein of the predicted size of 150 kDa in 3D7 parasites that was absent in ΔPfSir2B parasites (Figure 2D). We wished to compare the expression of *var* and other subtelomeric genes between wildtype and Sir2-disrupted lines, and given the high variation in these areas between isolates we confirmed that all of the derived parasite lines were isogenic by fingerprint analysis using the *rep20* repetitive probe (Figure S2) [42].

### PfSir2A and PfSir2B Regulate Silencing of Distinct Groups of *var* Genes in P. falciparum

To determine if PfSir2B, a putative histone deacetylase, has a role in gene regulation within the intraerythrocytic life cycle of P. falciparum we performed global transcription analysis with ΔPfSir2A, ΔPfSir2B, and the parental 3D7 strain using a custom-designed, 2.5-million feature Affymetrix chip based on the fully sequenced P. falciparum genome (3D7 strain version January 2005). Parasites were synchronised and RNA prepared from three time points within the ∼48-h intraerythrocytic lifecycle corresponding to: ring stage, 8–12 h post-invasion (pi); trophozoite stage, 24–28 h pi; and schizont stage, 38–42 h pi. Ring-stage parasites represent a time of host cell remodelling and expression of antigenically variant proteins that are exported to the P. falciparum-infected erythrocyte surface [43,44]. Trophozoites represent the period of growth and haemoglobin digestion, whereas schizonts define a period of daughter cell formation for subsequent egress and invasion into new erythrocytes. Three biological replicates for each stage and for all parasite lines were used for quality control. Similar variability between all samples demonstrated that no bias was introduced during the processing of parasites handling and sample preparation (unpublished data).

**Figure 3:**
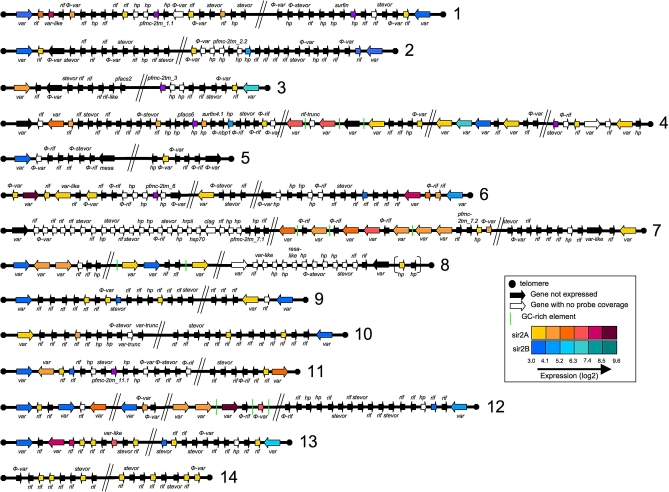
PfSir2A and PfSir2B Control the Expression of Subtelomeric Genes in P. falciparum The expression profile of subtelomeric genes and internal *var* gene clusters were analysed in Sir2-disrupted lines and applied to a physical map of all 28 chromosome ends. Subtelomeric ends contain most *var* genes as well as the *rifin* gene family, which are also known to be antigenically variant. Other gene families occupy subtelomeric regions of P. falciparum chromosomes, but at this stage their biological function or whether they are antigenically variant remains elusive. As seen by the key the colour of each gene relates to the level of expression in each PfSir2-disrupted line. Black genes indicate that expression of that gene is not observed in any condition (expression signal <75th percentile). In the unusual case where PfSir2A or PfSir2B both affected the expression of a single *var* gene only the most dramatic change is shown. This physical map of gene expression accompanies Figure S3, which graphs the expression values of all subtelomeric genes in 3D7 and the two PfSir2-disrupted lines.

The Sir2 protein in yeast affects the expression of subtelomeric genes, and the *P. falciparum* homologue PfSir2A has previously been implicated in the silencing of a subset of *var* genes in early ring stages [24]. Consequently we determined the transcriptional profile of subtelomeric genes within the P. falciparum genome in both ΔPfSir2A and ΔPfSir2B parasites. In ΔPfSir2A and ΔPfSir2B we physically mapped the pattern of gene expression within subtelomeric regions of all 14 P. falciparum chromosomes, and the five internal *var* gene clusters (Figure 3). Strikingly, we found that between the ΔPfSir2A and ΔPfSir2B parasite lines, essentially all *var* genes were transcribed. Also, it was evident that there was a correlation between the particular *pfsir2* gene disrupted and the physical position and direction of transcription of the activated gene (Figure 3). That is, primarily the most telomere proximal *var* genes (i.e., controlled by an UpsB promoter) were highly activated in ΔPfSir2B, whereas the subtelomeric *var* genes transcribed towards the telomere and those in internal chromosomal clusters (i.e., controlled by UpsA, UpsE, and UpsC) were highly transcribed in ΔPfSir2A (Figure 3, rainbow pattern of expression blue to green and yellow to purple, respectively, see also Figure S3). This suggests that PfSir2A plays an important role in silencing of UpsA, UpsC, and UpsE controlled *var* genes whilst PfSir2B silences the nonoverlapping group controlled by UpsB promoters.

### Analysis of *var* Gene Transcription in P. falciparum Parasites Lacking PfSir2A or PfSir2B Function

**Figure 4:**
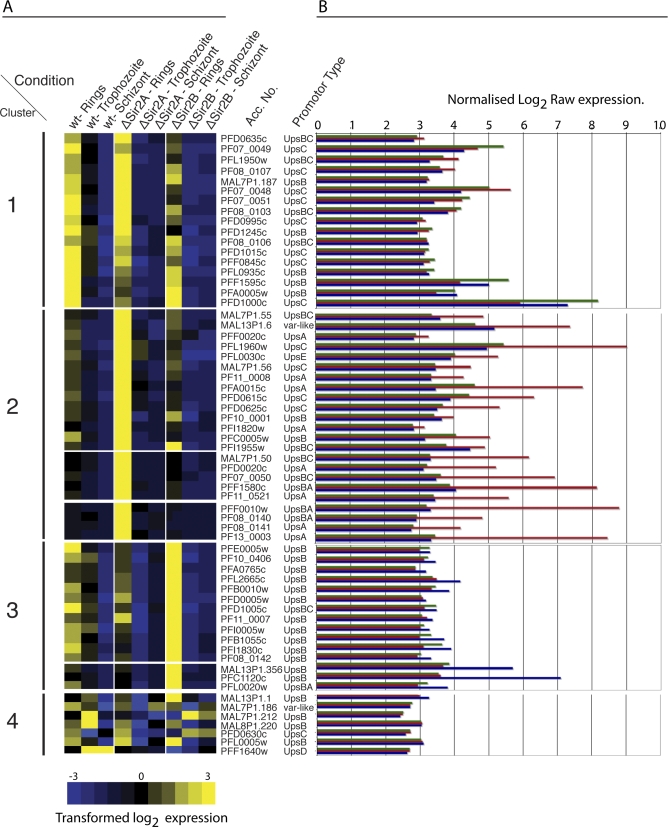
PfSir2 Paralogues Silence Discrete Subsets of *var* Genes Associated with Conserved Promoter Types That Drive Their Expression (A) Unsupervised nonhierarchical Gaussian self-clustering analysis was performed in order to understand the relationship between *var* gene subtypes and the role of PfSir2 paralogues in their regulation. Genes were clustered by similarity using Pearson coefficients. Heatmap colour-scale values were assigned by ArrayMiner after further transformation of expression data (see Material and Methods for details) with +3 (yellow) being the highest expression and −3 (blue) being the lowest expression. Expression profiles of the *var* gene repertoire were grouped given the nine conditions examined by Affymetrix arrays (i.e., rings, trophozoites, and schizonts in 3D7 [wildtype], ΔPfSir2A, and ΔPfSir2B parasite lines). The heatmap clearly identifies three major clusters that are biologically relevant. Cluster 1 represents *var* genes that are expressed in standard culture conditions in 3D7 parasite isolate and are somewhat expressed in PfSir2-disrupted parasites. No correlation in promoter type is seen in *var* genes found is this cluster with the exception that no UpsA-*var* genes were found there. *Var* genes controlled by UpsA conserved promoter type are found within cluster 2 as are most of the internally located UpsC *var* genes. This finding suggests that PfSir2A plays a suppressive role in expression of *var* genes with an UpsA or UpsC promoter type. Cluster 3 contains almost exclusively UpsB *var* genes and these are de-repressed in the absence of PfSir2B. This suggests that PfSir2B is involved in the suppression of the most telomere proximal, UpsB driven *var* genes. Cluster 4 contains several *var* genes that seem to be outliers, not conforming or reaching the threshold required to fall into one of the three main clusters. Scale bar of expression goes from −3 to 3 in transformed log_2_ expression. (B) Raw normalised log_2_ expression of all *var* genes in ring-stage parasites aligned with their corresponding heatmap in the Gaussian self-clustering analysis. Raw normalised expression values for all *var* genes grouped by their promotor type as assayed by array analysis can be viewed in Figure S4. The colour of each bar refers to its strain identity: green, 3D7; red, ΔPfSir2A; blue, ΔPfSir2B.

The expression of *var* genes was further analysed in wildtype and the two Sir2-disrupted lines. A Gaussian “self”-clustering analysis was performed on the transcription pattern of the full repertoire of *var* genes in the 3D7 genome given nine different conditions set out in our array experiments (i.e., three parasite lines; ΔPfSir2A, ΔPfSir2B, and 3D7 at three different time points; rings, trophozoites, and schizonts) (Figure 4A). *Var* genes were also labelled by their promoter type, UpsA, B, C, E, D, *var*-like, and hybrid promoters BC and BA [18]. Gaussian self-clustering grouped transcription profiles into three distinct clades with cluster 4 containing outliers (Figure 4A). Cluster 1 included *var* genes that are expressed in parental 3D7 during standard culture conditions and they were also expressed in ΔPfSir2A and ΔPfSir2B parasites (Figure 4A). There was no obvious bias towards the promoter type driving the expression of these *var* genes, nor could we find any other feature that defined this cluster except that no UpsA-driven *var* gene was found. This most likely represents the “stable” *var* gene transcription observed in the parental population of 3D7 in standard culture conditions that has not been selected for any cytoadherence phenotype.

Cluster 2 represents *var* genes that are poorly expressed in parental 3D7 but that are highly upregulated in ΔPfSir2A at ring stage suggesting a role for this molecule in silencing this subset of genes (Figure 4A). Interestingly, all *var* genes controlled by an UpsA promoter (subtelomeric and transcribed towards the telomere) are contained within this cluster (Figure 4A). This cluster also contains a significant proportion of UpsC promoter controlled *var* genes. UpsC promoters drive the expression of *var* genes that are found in internal chromosome locations, showing that PfSir2-mediated silencing is not restricted to subtelomeric genes (Figure 4A) and that PfSir2A is required for their silencing. Several *var* genes controlled by hybrid promoters, in which the 3′ end represents an UpsB promoter and the 5′ end has similarity to UpsC or UpsA promoters (i.e., UpsBC and UpsBA), are also found within this cluster, which suggests that a common feature of these hybrid *var* promotors allows their control by PfSir2A.

Cluster 3 includes *var* genes that are derepressed in the absence of PfSir2B and therefore suggests a role for this histone deacetylase in the silencing of this *var* gene subset (Figure 4A). Of the 15 *var* genes within this cluster, UpsB promoters control 13, the other two are hybrid promoters (UpsBA and UpsBC). Several UpsB *var* genes also show a high degree of statistical significance in transcription between parental and knockout (Figure S4) and these were confirmed by quantitative PCR (qPCR) (see below and Table S1). The remaining seven *var* genes do not fall into a distinct cluster (although here called cluster 4 in Figure 4A) owing to atypical expression patterns (MAL7P1.186, MAL7P1.212, MAL8P1.220, PFF1640w), but also perhaps because of the derepression phenotype not meeting the threshold for the Gaussian clustering (MAL13P1.1, PFL0005w) (Figure 4A). Figure 4B displays the normalised log_2_ raw expression data of all *var* genes, in rings, in a bar graph format as an alternative way to view the data. Individual *var* genes are aligned with the *var* genes as they appear in the Gaussian clustering analysis (Figure 4B). This data can also be viewed in an alternate way in supporting information as raw normalised expression of each *var* gene, grouped according to *var* promoter type in bar graph format (Figure S4). qPCR was used to confirm the Affymetrix array results (see below and Table S1). In conclusion, P. falciparum Sir2 paralogues play a role in silencing the *var* gene repertoire and there is a strong correlation between the *var* promoter type and the PfSir2 molecule that regulates its activity.

### PfSir2 Paralogues Control Mutual Exclusive Transcription of *var* Genes

**Figure 5:**
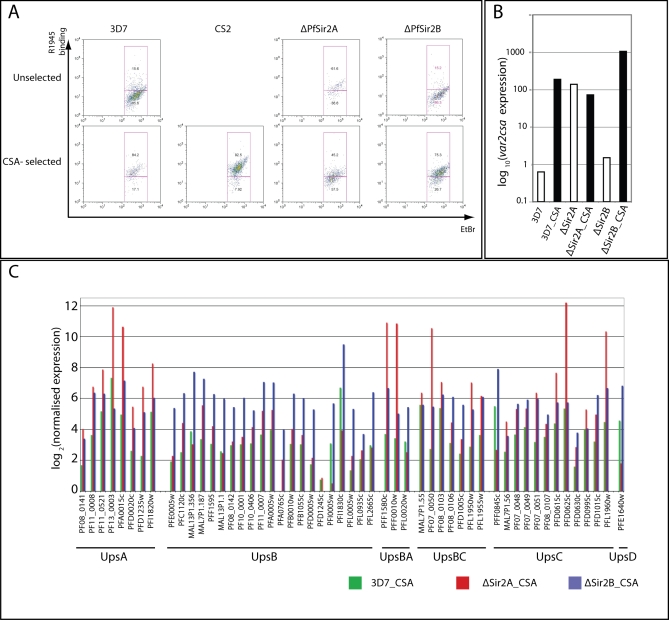
Disruption of PfSir2 Paralogues Destroys Mutual Exclusive Expression of *var* Genes In wildtype parasites selection on CSA enriches for parasites exclusively expressing *var2CSA*. We used this mono-allelic binding phenotype to assay if PfSir2-disrupted parasites still maintain mutual exclusive expression of *var* genes. (A) 2-D FACS plots showing binding of R1945 rabbit serum specifically recognises infected erythrocytes (ethidium bromide [EtBr] positive) containing *var2CSA*-expressing parasites. R1945 percentage positivity and negativity are separated on the basis of fluorescence levels of uninfected erythrocytes. Selection on CSA of 3D7 (3D7_CSA) greatly enhances the reactivity to R1945 serum and is comparable to the CS2 parasite line that stably expresses *var2CSA*. Given that ΔPfSir2A already expresses *var2CSA* in the absence of selection it is not surprising that CSA selection of this parasite line (ΔPfSir2A_CSA) does nothing (if anything a negative effect) to the binding of R1945 serum. ΔPfSir2A parasites show an intermediate binding of R1945 reactivity suggesting that they have lower levels of VAR2CSA on their surface. Selection of ΔPfSir2B parasites on CSA (ΔPfSir2B_CSA) also greatly enhanced R1945 binding to infected erythrocytes suggesting the ΔPfSir2B parasites are a heterogenous pool and are not confined to expressing only UpsB-driven *var* genes. (B) As expected qPCR of *var2CSA* (PFL0030c) transcript levels shows a dramatic increase upon CSA selection on 3D7 and ΔPfSir2B parasites, but not ΔPfSir2A. (C) Mutual exclusive expression of *var* genes is broken down in the absence of both PfSir2A and PfSir2B. As expected, 3D7_CSA mainly expresses *var2CSA* and while other *var* genes are only at a very low levels (see also Figure S5). However, when compared to 3D7_CSA, PfSir2-disrupted parasites, not only express *var2CSA* but also express many other *var* genes at much higher level than 3D7_CSA does (silenced state). This finding strongly suggests that mutual exclusive expression has broken down in these parasites. Interestingly, expression of *var* genes in the Sir2-disrupted lines selected on CSA also shows a bias towards *var* genes with certain promoter types thus confirming by another method our earlier findings.

Antigenic variation of PfEMP1 occurs by monoallelic activation of a single *var* gene and silencing of all others [13]. Switching between PfEMP1 variants occurs at approximately 1% per generation and this involves, through unknown mechanisms, silencing of one *var* gene and activation of another [45]. Given that we are analysing a population of parasites expressing a range of *var* genes it was not possible to conclude from our initial analysis whether the absence of PfSir2A and B function results in a greater frequency of switching or a breakdown in the mutually exclusive program by the derepression of multiple *var* genes in a single parasite. To discern between these possibilities parasites were selected for adherence to CSA, which is mediated by a PfEMP1 protein encoded by the *var2csa* gene (3D7 genome: PFL0030c) [10,46], and determined the transcriptional levels of the *var* gene repertoire. Selection of ΔPfSir2A and ΔPfSir2B parasites on CSA allowed us to determine the integrity of *var* gene mutual exclusion in a ΔPfSir2A and ΔPfSir2B genetic background (Figure 5).

3D7, ΔPfSir2A, and ΔPfSir2B were selected for high levels of CSA adherence to produce the lines 3D7_CSA, ΔPfSir2A_CSA, and ΔPfSir2B_CSA. These lines were firstly analysed by flow cytometry (FACS) for expression of VAR2CSA, the PfEMP1 protein responsible for CSA adherence [46], using antibodies that could specifically detect this protein (Figure 5A) [47]. 3D7_CSA parasite-infected erythrocytes expressed increased levels of VAR2CSA compared to 3D7 consistent with selection of parasites expressing this PfEMP1 protein (Figure 5A) [48]. The level of VAR2CSA expression for 3D7_CSA was comparable to CS2, a parasite line that more stably expresses this PfEMP1 variant (Figure 5A). The ΔPfSir2A parasites transcribe elevated levels of *var2csa* along with other *var* genes controlled by UpsA and E promoters [24], consequently it was not surprising that ΔPfSir2A_CSA parasites expressed similar levels of VAR2CSA on the surface of infected erythrocytes compared to ΔPfSir2A_CSA (Figure 5A). It should be noted that by scatter plot PfSir2A-disrupted parasites do not bind as much anti-VAR2CSA antibody suggesting that they have less VAR2CSA on their surface (Figure 5A). In contrast, ΔPfSir2B parasites do not express VAR2CSA as anti-VAR2CSA binding to the parasite-infected erythrocyte was low. However, upon CSA selection ΔPfSir2B_CSA-infected erythrocytes had high levels of anti-VAR2CSA (R1945) reactivity, suggesting these parasites retain the capacity to activate expression of other PfEMP1 antigenic types (Figure 5A).

To address the role of PfSir2A and B on *var* gene switching and monoallelic exclusion at the transcriptional level qPCR was performed with cDNA from 3D7, ΔPfSir2A, and ΔPfSir2B parasites before and after CSA selection to determine alterations in transcription of this gene family (Figure 5B and 5C; Table S1). As expected the *var2csa* gene in 3D7 was dramatically increased in transcription after selection in 3D7_CSA, which correlated with increased reactivity to VAR2CSA antibodies (Figure 5B) [49]. All other *var* genes were down regulated to apparently basal levels (Figures 5C and S5) consistent with an intact mutually exclusive *var* expression program. Given that ΔPfSir2A already expresses *var2csa* at high levels [24], selection on CSA did not substantially affect its level of gene transcription (Figure 5B). Interestingly, and in concordance with the anti-VAR2CSA antibody binding, selection of ΔPfSir2B parasites on CSA dramatically increases *var2csa* transcription in these parasites 5–10-fold compared to 3D7_CSA (Figure 5B). These results suggest that loss of PfSir2B function has not disrupted the ability of these parasites to activate specific silenced *var* genes.

To determine if the PfSir2 paralogues play a role in control of mutually exclusive expression, the transcription of *var* genes was analysed before and after selection for adherence to CSA (Figure 5C). Given that all *var* genes except *var2csa* are silenced in 3D7 parasites selected on CSA, this analysis was used as a benchmark for the *var* gene silenced state (Figures 5C and S5). Comparing the expression of *var* genes in ΔPfSir2-disrupted parasites selected on CSA with 3D7_CSA parasites it can be seen that in both ΔPfSir2A_CSA and ΔPfSir2B_CSA express multiple *var* genes at higher levels than 3D7_CSA. Indeed, some *var* genes in ΔPfSir2A and ΔPfSir2B are expressed higher than in 3D7 (Table S1). These data suggest that mutually exclusive expression of *var* genes has broken down in the absence of PfSir2 paralogues and thus both these molecules play a critical role in the control of mutually exclusive expression of *var* genes (Figure 5C). It is also worth noting that ΔPfSir2A_CSA and ΔPfSir2B_CSA infected erythrocytes can bind to CSA and CD36 and this together with anti-VAR2CSA antibody reactivity is consistent with expression of multiple PfEMP1 variants on individual infected erythrocytes and the breakdown of mutual exclusion (unpublished data). Both before and after selection of ΔPfSir2A and ΔPfSir2B for CSA adherence a similar pattern of *var* gene transcription was observed consistent with an important role for PfSir2A and PfSir2B in silencing of specific *var* gene subsets (Figure 5C; Table S1).

Using this type of analysis it also looks as though PfSir2A and PfSir2B can have an effect on the transcription of the same *var* gene suggesting that they are acting cooperatively rather than exclusively (Figure 5C). For example, UpsB-driven *var* genes are largely regulated by PfSir2B, however, using qPCR and CSA selection PfSir2A clearly has an effect on UpsB controlled gene transcription (greater log_2_ value) (Figure 5C). The reciprocal is true for UpsA and UpsC *var* genes where parasites lacking PfSir2B function transcribe greater levels of UpsA *var* genes compared to the same genes silenced in 3D7 parasites. This result suggests that PfSir2 paralogues act cooperatively to control the transcription of individual *var* genes and warranted further investigation.

### PfSir2 Paralogues Have a Direct Effect on the Silencing of *var* Gene Promoters

**Figure 6:**
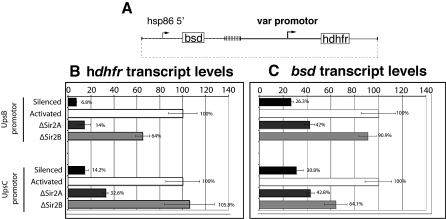
PfSir2 Paralogues Directly Regulate *var* Gene Promoters and Contribute to Spreading of Transcriptional Silencing into Neighbouring Regions (A) Plasmids bearing two expression cassettes were introduced into 3D7, ΔPfSir2A, and ΔPfSir2B parasites. The *bsd* cassette is driven by the housekeeping *hsp86* promoter, which provides a selection mechanism for parasites stably maintaining episomes. UpsB and UpsC *var* promoters were placed in front of the selectable marker *hdhfr* and therefore *var* promoter activity can be monitored. All values plotted were normalised against the *var* promoter in its activated state. (B) PfSir2A and B paralogues differentially affect the silencing of isolated *var* promoters. *hdhfr* transcript level was measured in 3D7 parasites with silenced and activated episomally located UpsB and UpsC promoters as well as the level of *hdhfr* transcription in the absence of PfSir2 paralogues. Both PfSir2 paralogues can directly affect the activity of a *var* promoter strongly suggesting that PfSir2 paralogues do not function independently of one another on different *var* promoters but rather cooperate to regulate expression. In both subtelomeric and internally derived *var* promoters isolated from their natural context PfSir2B has a larger effect on the suppression of *var* promoter activity. (C) PfSir2A and PfSir2B control the spreading of silencing from *var* promoters. Upon activation of the downstream *var* promoter activity of *bsd* expression cassette increases suggesting that factors that normally silence *var* gene promoters can spread in *cis* to modulate expression of neighbouring regions. In the absence of both PfSir2A and PfSir2B spreading of silencing is reduced suggesting that both paralogues mediate the spreading of silencing from both internal and subtelomerically derived *var* gene promoters. In both cases PfSir2B has a greater influence on spreading of silencing to within the *bsd* cassette. qPCR was performed on three biological replicates harvested from ring-stage parasites. 95% confidence error bars derived from a calculated standard error are also displayed.

Previously, we showed that the default state of a *var* promoter is silenced and stochastic activation occurs at low frequency [26,27]. Additionally, a drug resistance marker can be inserted into the *var* gene antigenic program by placing it under the control of a *var* promoter so that its activation by drug selection enforces silencing of all *var* genes [26,27]. To investigate whether PfSir2 paralogues directly regulate the silencing of *var* genes by acting on the promoter we introduced plasmids with an UpsB (PFL0005w; subtelomeric) or UpsC (PFL1960w; internal) *var* promoter driving *hdhfr* in 3D7, ΔPfSir2A, and ΔPfSir2B parasites and assayed promoter activity by qPCR. Plasmids also contained the blasticidin-S deaminase (*bsd*) gene driven by the constitutive *hsp86* promoter for selection of transfected parasites (pUpsB/hDHFR and pUpsC/hDHFR) (Figure 6A).

In 3D7-pUpsB/hDHFR and 3D7-pUpsC/hDHFR, selection with the antifolate WR99210 provided a parasite population expressing activated versions of the UpsB and UpsC promoters respectively [26,27]. As expected *hdhfr* gene transcript levels (after normalising for plasmids copy number) are dramatically increased compared to WR99210 unselected lines (Figure 6B). ΔPfSir2A and ΔPfSir2B parasites containing either pUpsB/hDHFR or pUpsC/hDHFR transfected parasites could not be selected on WR99210 because *h*DHFR (and WR99210 selection) was used to make the *PfSir2* gene disruptions. Consequently *var* promoter activity was determined in the default state using qPCR.

Analysis of the UpsB and UpsC *var* promoter activity in ΔPfSir2A-pUps(B/C)/hDHFR and ΔPfSir2B-pUps(B/C)/hDHFR (after subtracting the transcript signal of *hDHFR* from ΔPfSir2A and B lines) showed that absence of PfSir2A and B function results in a partial derepression of the *hdhfr* gene (Figure 6B). These results demonstrate that PfSir2-mediated silencing can act directly on *var* promoters and does not require *cis-*spreading of silenced chromatin from neighbouring telomeres as is observed in Sir2-mediated subtelomeric silencing in yeast [50]. Interestingly, this analysis shows that both Sir2 paralogues play a role in the silencing of individual *var* promoters, as first observed earlier in qPCR of *var* genes in PfSir2-disrupted lines selected on CSA (Figure 5C). Interestingly, loss of either PfSir2A or B function causes only partial derepression compared to a fully activated *var* gene promoter, strengthening the idea that both molecules cooperate in silencing these *var* promoters. The absence of PfSir2B shows greater activation of the isolated UpsB *var* promoter (PFL0005w), strongly supporting the evidence that PfSir2B plays a greater role in the regulation of UpsB *var* genes than does PfSir2A. In the case of the UpsC *var* promoter (PFL1960w) there is a greater derepression of this promoter in the absence of PfSir2B, which is somewhat contradictory to the *var* qPCR and microarray analysis of the endogenous PFL1960w gene in all the cell lines (Figure 6B). One possible explanation for this is slight differences in the complex regulatory network of *var* genes when they are located in different chromosomal contexts (i.e., internal chromosome location versus extrachromosomal episome). *Var* gene promotors are key to the control of mutual exclusive expression of *var* genes, and our results strongly implicate PfSir2 paralogues as major *trans*-acting factors influencing the control of these highly conserved elements. This clearly highlights the importance of PfSir2 paralogues in the control of *var* gene silencing and mutual exclusion.

### Spreading of Silencing Is Mediated by PfSir2 Paralogues

The spreading of heterochromatin from its nucleation point is important to achieve gene and regional silencing [51]. Previously we have shown that silencing can spread from a *var* promotor [26,27]. Sir2 in yeast is known to be involved in spreading of silencing chromatin [50], and we reasoned that PfSir2A and B could also be involved in this process in P. falciparum. We investigated whether PfSir2 paralogues had a role in spreading of silencing by monitoring the expression level of a *bsd* expression cassette upstream of a *var* promotor (Figure 6A). We assayed the transcript level of *bsd* (driven by the housekeeping *hsp86* promoter) by qPCR in 3D7, ΔPfSir2A, and ΔPfSir2B parasites containing pUps(B/C)hDHFR plasmids (Figure 6C). Parasites with an active, plasmid-based UpsB or UpsC promoter driving the *hDHFR* gene in 3D7 had significantly increased levels of the *bsd* transcript, suggesting spreading of *var* gene silencing factors to the *hsp86* promoter, as has been observed previously [26]. Interestingly, upon introduction of pUpsB/hDHFR into ΔPfSir2A and B parasites the activation state of the *bsd* cassette differed from that of parental 3D7 parasites with a silenced, plasmid-located *var* promoter (Figure 6C). As observed with the activation of *var* promoter in Sir2-disrupted lines, the effect on the upstream *bsd* cassette differed depending on which PfSir2 paralogue was not functional. In the case of UpsB-based plasmid we found an almost complete abrogation of silencing in the PfSir2B-disrupted line and a more mild alleviation of silencing in parasites lacking PfSir2A (Figure 6C). A similar phenomenon was observed with UpsC-based plasmids, suggesting that spreading of silencing is less reliant on the function of PfSir2B (Figure 6C). These data strongly suggest that both PfSir2 molecules, to differing degrees, play a role in the spreading of a transcriptionally repressed state from *var* promoters.

### Temporal and Transcriptional Regulation of the Variant *rif* Gene Family Is Altered by Loss of PfSir2B or PfSir2A Function

**Figure 7:**
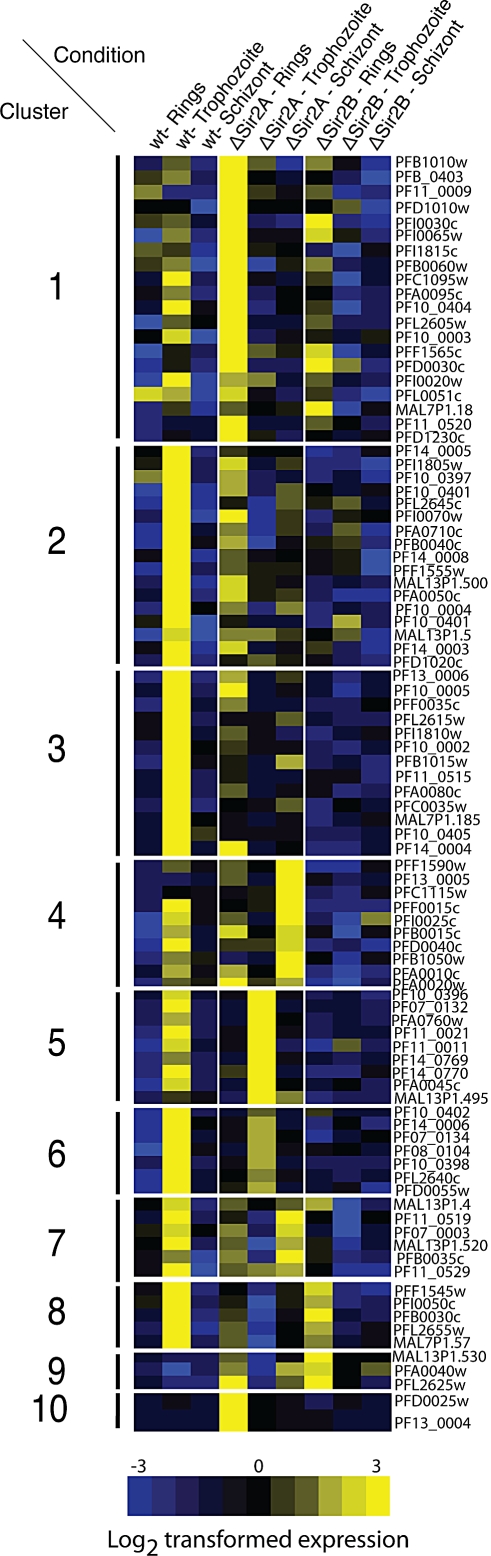
*rif* Genes Change Expression Timing upon Disruption of PfSir2A, but not PfSir2B Gaussian self-clustering was used to further analyse the role of PfSir2 paralogues in *rif* gene expression. Expression throughout rings, trophozoite, and schizont-stage parasites show that PfSir2A can modulate the temporal expression of *rif* genes. Normally most *rif* are expressed in trophozoite stage but upon disruption of PfSir2A expression of some of this antigenically variant gene family changes to either ring- or schizont stage, or both. Interestingly the lack of PfSir2B shows a suppressive effect on rif gene expression. Unfortunately no other similarities either in promoter sequence or physical position relative to *var* genes could be found in any of the clusters.

Repetitive interdispersed family (*rif*) genes constitute the largest gene family in P. falciparum consisting of upwards of 150 members, most of which are within subtelomeric regions [10,52]. Other than *var, rif* genes are the only other gene family to date that has been shown to be antigenically variant [52] and are normally expressed in trophozoite-stage parasites [52]. The encoded Rifin proteins are trafficked to the infected erythrocyte, and some data suggest that they are located on the red blood cell surface [52]. By analysing microarrays we found that a proportion of the *rif* gene family was activated in ΔPfSir2A and ΔPfSir2B parasites and these often have a close physical association with an activated *var* gene (Figure 3) (see below for further analysis). Gaussian self-clustering was performed on the *rif* genes with respect to the nine conditions under which the microarray analysis was performed (Figure 7). Ninety *rif* genes sorted into ten different clusters, whilst the remainder were not transcribed under any of the conditions and were therefore excluded from the analysis.

Analysis of *rif* transcription across the nine conditions demonstrated differences between both ΔPfSir2A and B parasites (Figure 7). It was apparent that ΔPfSir2A parasites contain changes in raw transcriptional levels of *rif* genes compared to parental, as was observed with *var* genes, however, a shift in transcriptional timing was dramatically evident accounting for most of the variation observed compared with 3D7 parasites (Figure 7). Lack of PfSir2A function results in alteration of the temporal regulation for *rif* gene transcription from trophozoites to ring stage (clusters 1, 9, and 10) and in some cases also to schizont stage (clusters 4 and 7). Comparison between 3D7 and ΔPfSir2A parasites showed that *rif* genes in clusters 1, 2, 3, and 8 in ΔPfSir2A were expressed at ring stages, whereas clusters 4 and 7 were expressed mainly at schizont stages, even though some transcription can still be accounted for at ring-stage parasites (Figure 7). Clusters 5, 6, 9, and 10 showed slightly different variations. While the *rif* genes in clusters 5 and 6 remain expressed at the trophozoites stage, their level of transcription varied in ΔPfSir2A trophozoites and was higher in cluster 5 but lower in cluster 6 (Figure 7). For the *rif* genes classified in clusters 9 and 10, whilst they showed no detectable transcription in parental 3D7, they were activated in ΔPfSir2A and also showing a shift in their temporal pattern (Figure 7). Interestingly, ΔPfSir2B parasites do not transcribe any *rif* genes at the schizonts stages suggesting that loss of PfSir2B function results in repression of this gene family (Figure 7). Transcription of some *rif* genes showed a shift towards ring stages as was found for ΔPfSir2A parasites but had a decreased level of transcription when compared to the 3D7 parent (Figure 7, clusters 1, 8, and 9).

We attempted to find features common to the *rif* genes found in each individual cluster, similar to the *var* gene analysis showing that the conserved promoter types also fall into distinct clusters. *Rif* promoters are also conserved [53], and the encoded *rifin* genes have been phylogenetically classified into two groups [54]. We performed phylogenetic analysis on the 1.5-kb promoter regions of the *rif* genes in Figure 7, and the cluster number was mapped to each *rif* gene within the phylogenetic tree. We found no correlation of relatedness between promoter sequence and the likelihood of being expressed in either PfSir2-disrupted line, suggesting that unlike *var* genes the *rif* promoter does not have a prominent role with respect to regulation of this gene set by PfSir2A or B. Additionally, there was no strong correlation between the physical positioning of *rif* genes compared to *var* genes other than the strongest expressed *rif* transcripts are found head-to-head with expressed *var* genes in ΔPfSir2A parasites (unpublished data). These results show that loss of PfSir2A or B function has a strong effect on a proportion of the *rif* genes suggesting they are important in the regulation of this variant gene family.

### The Role of PfSir2A and PfSir2B in Regulation of Other Genes in P. falciparum

Transcriptional analysis of the ΔPfSir2A and ΔPfSir2B parasites showed that PfSir2A and B proteins played an important role in silencing of *var* genes and regulation of the *rif* gene family, but surprisingly had virtually no effect on other genes within the P. falciparum genome with some notable exceptions. A small number of genes other than *var* and *rif* located within the subtelomeric region of chromosomes showed increased transcription in ΔPfSir2A, which included the *pfmc-2tm* gene family [55]. This family consists of 12 members and ten are transcribed in the 3D7 parent (unpublished data). Removal of PfSir2A function in ΔPfSir2A resulted in increased transcription of the ten genes that were already transcribed to some extent (unpublished data). Figure S3 displays the physical map of subtelomeric genes and internal *var* gene loci with accompanying expression graphs for each gene throughout the intraerythrocytic time points applied to the array.

After removing all the *var* and *rif* genes from the analysis we scanned the remaining differentially expressed genes for common features (Table S2). Interestingly, we found that most differentially expressed genes found in either *ΔPf*Sir2A or B was transcribed in ring-stage parasites. We also noticed that PfSir2A disruption affected expression of more genes compared to loss of PfSir2B function. Members of several other gene families changed expression profile in ΔPfSir2A and B lines, including several members of the large 72-member *Plasmodium* helical interdispersed subtelomeric (PHIST) family [43,56], a *stevor* gene, and an *etramp* (Table S2) [57]. The differentially expressed genes were compared with respect to their features and physical position along the chromosome and no obvious shared properties were identified (Table S2). We also specifically looked for other genes known to confer parasite phenotypic variation, and the expression of rDNA genes (which are affected by Sir2 in yeast) and we found no differences. For more in depth analysis please consult Text S1.

### PfSir2A Plays a Role in Maintenance of Telomere Length

**Figure 8:**
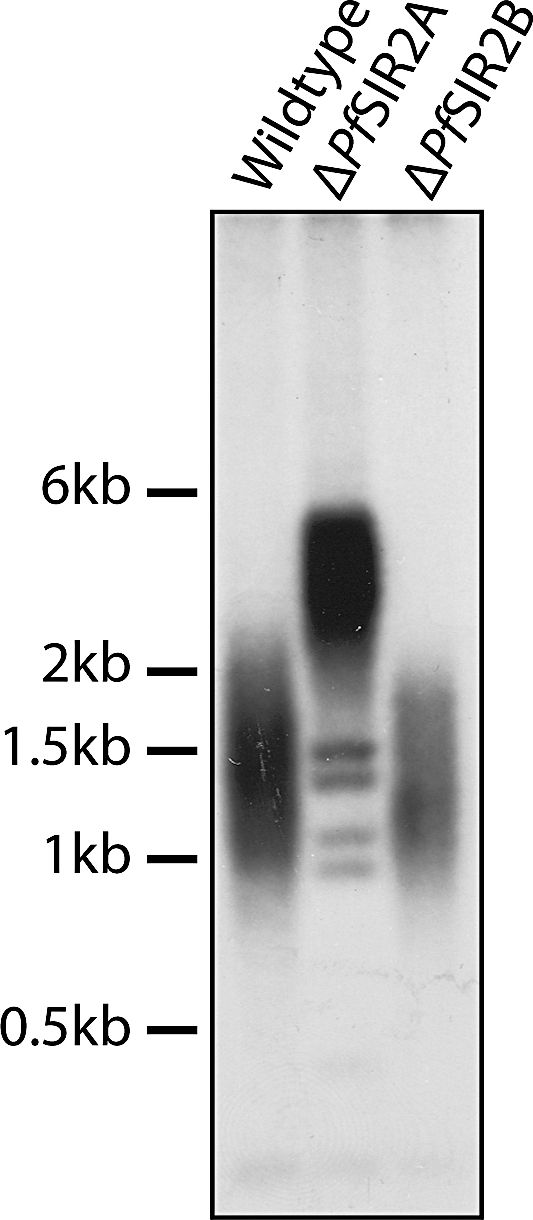
Sir2A, but Not PfSir2B Has a Role in Telomere Length Regulation Genomic DNA from wildtype (3D7), ΔPfSir2A, and ΔPfSir2B was digested with four frequently cutting restriction enzymes that do not cut telomeric repeats. Samples were run on 1% agarose gel and probed with telomeric repeat-labelled with ^32^P. Telomere length is seen as a smear as telomere lengths vary between chromosomes and within a population telomere. 3D7 parasites have telomere lengths of between 1–2 kB. Whereas upon disruption of PfSir2A but not PfSir2B, telomere length approximately doubles in size. PfSir2B-disrupted parasites have several distinct bands of a length that correspond to nonlengthened telomeres. It is also possible that they represent telomeric repeats that have recombined into chromosome internal locations and thus are not able to lengthen.

Telomeres recruit proteins that facilitate spreading of heterochromatin into subtelomeric noncoding and coding regions [58]. In P. falciparum, PfSir2 and PfORC1 associate with telomere and subtelomeric heterochromatin [39]. It has been shown in other systems that telomere length is influenced by factors that affect the chromatin fibre, including Sir3 and Sir4 from yeast [59]. Thus, it has been speculated that these factors are involved in telomere end protection. We reasoned that PfSir2 paralogues might be involved in this process. To assay the effect of PfSir2 paralogues on maintenance of telomere length we performed telomere restriction fragment (TRF) analysis as described previously [22]. This technique takes advantage of the lack of restriction sites within telomeric repeat DNA as a means to liberate intact telomeres. Southern blot on genomic DNA digested from 3D7, ΔPfSir2A, and ΔPfSir2B with four restriction enzymes that cut frequently within the P. falciparum genome but do not cut in telomeric repeats were probed with telomeric repeats (Figure 8). In a population of wildtype (3D7) parasites telomere repeat length varies from ∼1 kb to ∼2 kb over all chromosomes with a mean length of approximately 1.5 kb. In parasites lacking PfSir2A, but not PfSir2B an increase in telomere length is evident with an increase in mean length of 3 kb in length, approximately double the length of telomeric repeats in wildtype (3D7) and ΔPfSir2B parasites (Figure 8). It is also interesting to note that four sharp bands are evident in parasites lacking ΔPfSir2A in the range of 1 to 1.5 kb (Figure 8). At this stage it is uncertain what these bands represent in the chromosomal context. Given the fuzzy nature of chromosome ends due to variable number of telomere repeats, a likely explanation is that significant blocks of repeats have been moved to internal chromosome regions, leading to defined bands after TRF analysis. Thus, PfSir2A appears to increase chromosome end instability. This is supported by the Rep20 RFLP analysis (arrow, Figure S1, which indicates possible changes in the RFLP profile compared to 3D7 and ΔPfSir2A.) Interestingly, we noticed that there are four chromosome ends that do not contain a functional *var* most proximal to the telomeric repeats: left arm of Chromosome 6 (psuedo-*var*), the right arm of Chromosome 8 (hypothetical genes), and the two arms of Chromosome 14 (pseudo-*var*s) (Figure 3). This finding suggests a link between *var* gene regulation and telomere length by PfSir2 paralogues.

## Discussion

Antigenically variant PfEMP1 molecules are encoded by the *var* gene family and transcribed in a mutually exclusive fashion and controlled by epigenetic mechanisms [23,24,60]. We have shown here that two paralogues of the Sir2 NAD^+^-dependent histone deacetylase family cooperate to achieve silencing of the full *var* gene repertoire. This is, to our knowledge, the first demonstration of paralogous epigenetic modifiers cooperating to control the expression of a gene family and the first evidence of cooperation by Sir2 paralogous in the control of mutual exclusion of pathogen virulence factors.

The PfSir2 paralogues have separate but somewhat overlapping functions, and although both proteins have an effect on transcriptional repression of individual *var* genes PfSir2A has a greater role in silencing the subtelomeric UpsA promoter-driven genes (which are transcribed towards the telomeres) and the chromosomal internally located UpsC driven *var* genes [10]. PfSir2B, on the other hand, has a more prominent role in the silencing of *var* genes controlled by the subtelomerically located UpsB promoter type (which are transcribed towards the centromere). Both PfSir2A and B regulate *var* gene transcription by acting directly on the conserved promoters, which agrees with previous results analysing *var* promoters [26,30]. It appears likely that DNA elements within the conserved promoter types are a point that initiates *var* gene promoter silencing and spreading by recruitment of protein complexes containing either PfSir2A or PfSir2B. DNA elements that bind nuclear proteins have been experimentally identified in the different *var* promoter subsets [30]. The DNA element SPE2 was defined in UpsB promoters and mediates repression throughout ring and trophozoite-stage parasites when inserted into the housekeeping calmodulin promoter [27]. This DNA element is a candidate for initiating PfSir2B-mediated suppression in UpsB promoters by recruitment of proteins for heterochromatin formation and spreading of silencing.

*Var* promoters were analysed in an attempt to define a region that may be involved in initiating binding of PfSir2-containing complexes by using the hybrid UpsBA and UpsBC promoters that are regulated differentially by PfSir2A and B. The 3′ end of these promoters represents an UpsB-type promoter, whilst the 5′ resembles either an UpsA or C promoter generated by recombination between the different *var* gene subsets [18]. By utilizing the breakpoint in homology between these hybrid promoters we attempted to identify candidate regions of the promoter that could bind and initiate PfSir2-mediated silencing. However, we did not find any obvious conserved DNA elements in the promoter regions and it is likely the sequences involved are not well conserved. It will require further analyses using chromatin immunoprecipitation (ChIP) with PfSir2-specific antibodies and functional assay of the *var* promoter regions to identify these elements. Unfortunately the antibodies described here were not specific enough to address this question.

Both PfSir2 paralogues are required for mutually exclusive expression of *var* genes because ΔPfSir2A and ΔPfSir2B parasites express multiple genes and PfEMP1 proteins. Previous work has suggested that an active *var* gene is located in a specific subnuclear expression site at the nuclear periphery [11,24,25], and a candidate region is located at the nuclear periphery that lacks the electron dense appearance of heterochromatin [11]. The telomeric regions of chromosomes form clusters of 3–7 chromosome ends attached to the nuclear periphery, and in order for a *var* gene to be active it moves either to an “active” cluster [24] or outside a cluster to a transcriptionally permissive domain [11]. The UpsC *var* genes, that are more centrally located, also appear to be located within clusters at the nuclear periphery consistent with this area being important for both silencing and activation of this gene family [11]. Telomeric clusters are formed in the absence of PfSir2A [16] and PfSir2B (A. Marty, B.S. Crabb, and A.F. Cowman, unpublished data) function, and thus these proteins are not important for formation and maintenance of these chromosomal structures. It is not clear how parasites lacking PfSir2A or B function are able to accommodate transcription of multiple *var* genes once monoallelic exclusion has broken down, as these active genes must be located at the nuclear periphery. It is possible that loss of PfSir2A or B function results in breakdown of the subnuclear architecture spreading the transcriptionally permissive area or alternatively that multiple *var* genes can be transcribed within the existing permissive domain.

Both PfSir2A and B have a role in *var* regulation by acting directly on specific *var* promoter subsets and mediating spreading of the silenced state, which is in agreement with previous data [26,27]. This result is in contrast to studies in yeast where subtelomeric gene silencing is reliant on the spreading of silenced chromatin from the telomeric repeats and not direct binding to promoter regions (see for review [51]). Mechanistically, yeast subtelomeric transcriptional silencing is reliant on the spreading of silenced chromatin from telomeres and is mediated by the histone deacetylase activity of Sir2 [50]. At a molecular level this is thought to occur by the binding of RAP1 to the telomeric repeats, binding of the SIR complex to RAP1, followed by deacetylation of adjacent N-terminal Histone H3 and H4 tails. Subsequent binding of the SIR complex to the deacetylated histone tails then allows for deacetylation of adjacent histone tails and it is this iterative process that propagates the spreading of a silenced chromatin state inward into subtelomeric regions [50]. The apparent lack of RAP1 and SIR homologues in any of the sequenced apicomplexan genomes (www.plasmodb.org) [10] together with results presented here, suggest that this model is unlikely in P. falciparum. Rather our data support a model in which complexes containing either PfSir2A or PfSir2B can directly bind *var* promoter regions, regulating their expression and furthermore, spread silent chromatin in *cis*.

Subtelomeric regions of chromosomes in a variety of organisms are associated with epigenetic gene regulation [61]. Acetylation of H3 and H4 histone tails are associated with euchromatin and active gene transcription while the removal of these marks by histone deacetylases is linked to gene silencing and conversion of nucleosomes into a heterochromatic state [62]. In yeast Sir2, a specialised NAD^+^-dependent lysine histone deacetylase is found that suppresses the activity of subtelomeric genes [36]. Recently in P. falciparum it has been shown that histone H3 lysine 9 (H3K9ac) and acetylated histone H4 marks an active UpsE (related to UpsA) *var* promoter, while the PfSir2A homologue we also describe here binds to these promoter regions in P. falciparum [23,31]. Furthermore, we have shown here and elsewhere that PfSir2A is involved in its suppression [24].

PfSir2B also has a role in subtelomeric *var* gene silencing but mechanistically and biochemically it is not clear how this takes place. Although there is a dramatic size difference in the P. falciparum Sir2 paralogues, they share common features of the catalytic domains found in sirtuin homologues that have been studied structurally [63]. Interestingly, PfSir2B shows differences in its substrate-binding pocket suggesting these molecules bind different cellular targets. PfSir2A has previously been shown to localise to the nuclear periphery and associate with silenced, but not active *var* genes [23]. Furthermore, PfSir2A can deacetylate H3 and H4 histone tails and also ribosylate [38]. Given that PfSir2A and PfSir2B have different functions and contain differences in their substrate-binding pocket, it is likely that their cellular substrates differ.

It was surprising that there were so few genes affected by loss of PfSir2A or B function with the biggest effect being contained within the *var* and *rif* variant families suggesting epigenetic regulation may not be widely relevant to other genes in the asexual stages. PfSir2A, and to a lesser extent PfSir2B, has an effect on the level and temporal expression of the *rif* gene family. *Rif* genes sometimes have close physical proximity to *var* genes and have highly conserved promoters [10]. However, neither of these features correlated with derepression patterns in the absence of the PfSir2 paralogues. It appears that spreading of silenced chromatin from *var* genes is not a major factor in activation or alteration of temporal transcription of this gene family. The small number of genes affected by PfSir2A or B, other than *var* and *rif*, have no common features except that some are located within the subtelomeric region of the chromosomes. It will be interesting to determine if they have any common functional region within their respective promoters that render them sensitive to the PfSir2 proteins.

Telomeres are special nucleoprotein structures that protect chromosome ends from recombination and degradation. In most eukaryotes telomeres are assembled into tightly packed chromatin fibre [64]. Telomeres of P. falciparum form nucleosome and non-nucleosomal chromatin at the chromosome [65]. The biology of P. falciparum telomeres remains elusive apart from the characterisation of an unusually large telomerase subunit (TERT) [66,67]. No other *trans*-acting factors have been characterised that are required for telomere stability or length regulation. It has been shown that telomere length changes occur at truncated chromosomes in P. falciparum and this DNA sequence effect occurs only in *cis* [22]. Here we have identified PfSir2A, a heterochromatin component, that not only contributes to gene repression but affects telomere length at chromosome ends.

In the ΔPfSir2A parasites four DNA bands of sizes corresponding to unlengthened telomeres were present either corresponding to a subset of telomeres not lengthening in the absence of PfSir2A function or telomeric repeats that have recombined to internal locations. It was also evident using the rep20 RFLP analysis (a measure of subtelomeric integrity) that there were some differences in the PfSir2A-disrupted line when compared to wildtype parasites suggesting a PfSir2A role in chromosome stability. It is also interesting to note that there are four chromosome arm ends that do not have a functional *var* gene proximal [10] to the subtelomeric repeats drawing a possible link between *var* gene and telomere length regulation. It is also worth noting that in other systems telomere length is linked to genomic instability [68], and our data suggest also that this might also be the case for P. falciparum.

In summary, we have characterised the role of two Sir2 NAD^+^-dependent histone deacetylases on *var* gene regulation and show an important role for both paralogues in regulating antigenic variation through mutually exclusive transcription of the full *var* gene repertoire. The PfSir2 paralogues interact directly with the conserved *var* promoter subsets and have overlapping but distinct roles. It is not clear why P. falciparum parasites require the interplay of two PfSir2 paralogues for silencing of this important gene family. UpsA promoters control *var* genes that contain unusual cytoadherance-binding phenotypes, and studies suggest that their expression is associated with disease, whereas other *var* genes are associated with asymptomatic infections [69,70]. If these findings are confirmed, then perhaps PfSir2A has a role in suppressing the exposure of this limited repertoire of unusual cytoadherance phenotypes, and PfSir2B acts more as a general mediator of silencing and protection of the mutually exclusive program. Whatever the reason, given the clear importance of PfEMP1 in antigenic variation and cytoadherance, PfSir2 paralogues and other unidentified epigenetic factors are key determinants of pathogenicity in malaria infection.

## Materials and Methods

### Phylogenetic analysis.

Sequences were aligned using ClustalX v1.83 and alignments were manually adjusted using BioEdit Alignment editor. Phylogenetic trees were inferred from 253 positions of the alignments using both maximum likelihood and distance methods. Distance trees were made using both Neighbor joining and Kitsch methods using the PHYLIP 3.67 package. Distance bootstrapping was carried out for 1,000 replicates using the Seqboot bootstrapping method in PHYLIP 3.67 [71]. Maximum likelihood trees were made using PhyML version 2.5 [72] at http://www.phylogeny.fr/phylo_cgi/phyml.cgi, with the Whelan and Goldman (WAG) substitution model. Bootstrapping was performed for 100 replicates. In addition to the trees depicted, phylogenies were inferred from several alignment variants, including or excluding taxa or using fewer or greater alignment characters. Most nodes were insensitive to these variants, but the positioning and order of group III sirtuins varied with different datasets, indicating that this may not be a robust grouping.

### Cloning, transfection, and culturing.

Parasites lacking PfSir2B were created by the electroporation and selection for homologous integration of a bacterially derived plasmid harbouring a 5′ flank of the genomic sequence of the PfSir2B gene. The first 1,023 bp (including ATG) of the PfSir2B gene was amplified from 3D7 gDNA and ligated into the BglII/XhoI sites of pHHM (primer sequences available on request) [21]. Transfection and selection proceeded as previously described [73,74]. Cycling on and off drug selected for a population of parasites in which the plasmid has integrated via single crossover homologous recombination into the endogenous PfSir2B locus. Parasites were then cloned out by limiting dilution.

For investigations into the direct role that PfSir2 paralogues play on *var* promoter silencing and spreading, pHBUpsB [27] and pHBUpsC were introduced into wildtype (3D7), Δ*Pf*Sir2A, and Δ*Pf*Sir2B parasites and selected for stable maintenance with blasticidin-S. Wildtype parasites harbouring either plasmid were then also treated on WR99210 for selection of parasites expressing the ectopic UpsB promoter.

### Northern blotting.

Trophozoite-stage parasites were harvested using ice-cold saponin and resuspended in TRIzol (Invitrogen). Northern blots were performed as previously described [75]. Blots were probed with [^32^P]-dATP radiolabelled DNA and washed at 60 °C with 0.5 × SSC and 0.1% SDS prior to autoradiography. The ∼500-bp PfSir2B probe was amplified from within the flank used for the knockout construct (same as for southern blot).

### Southern blotting.

Southern blot analysis on ΔPfSir2B parasites was performed using standard procedures. gDNA was cut using BglII and runout on a 0.8% agarose gel. After transfer onto nitrocellulose a ∼500-bp fragment from the homologous flank containing incorporated dioxygenin-dATP was used as a probe (Roche). Primers for the amplification of the probe are available on request. Probe was detected on the membrane using anti-DIG-HRP antibodies and chemoluminescence detection (Roche).

Telomere length blot was performed as previously described [22]. Briefly, gDNA from 3D7, ΔSir2A(3D7), and ΔSir2B(3D7) was harvested from mixed-stage parasites. 2 μg of gDNA was cut with the four enzymes AluI, DdeI, MboII, and RsaI, which frequently cut within the *Plasmodium* genome, but not within the telomeric repeats. gDNA was run out on a 1% gel and transferred to Hybond N+ nitrocellulose membrane (Amersham). Membranes were probed with two complementary 99-mer oligonucleotides corresponding to nucleotides 11–110 of the left arm of Chromosome 13 (oligonucleotide sequences available on request).

### Antibody production.

DNA corresponding to amino acids 811–953 of PfSir2B was amplified from 3D7 gDNA and ligated into pGEX-4T1. Purified, bacterially expressed GST-PfSir2B 3′ was used to immunize rabbits. Before use, rabbit antiserum was purified over Protein-G sepharose (Amersham).

### Global expression analysis.

To analyse global gene expression profiles within intraerythrocytic cycles RNA was collected from highly synchronous cultures of ring-, trophozoite-, and schizont-stage parasites. Briefly, 3D7, ΔPfSir2A, and ΔPfSir2B parasites were tightly synchronised with 5% sorbitol twice 16 h apart. Parasites were harvested at 8–12 h, 24–28 h, and 38–42 h post invasion. Infected red blood cell pellets were immediately resuspended in TRIzol (Invitrogen) and frozen at −80 °C. RNA was extracted as per manufacturers protocol (Invitrogen) and further digested with RNase free DNase (Ambion).

*P. falciparum (Pf)* samples were hybridised on the custom PFSANGER Affymetrix array. PFSANGER Affymetrix arrays are high-density 8-μm custom 25-mer oligonucleotide arrays, whose tiling-like design was based on the P. falciparum (3D7 strain) genomic sequence released in January 2005 (www.genedb.org) [76]. 10 μg of total RNA was reverse transcribed and biotin-labelled as cRNA, using the GeneChip IVT Labelling kit as recommended by Affymetrix. Hybridisations were carried out at 45 °C for 16 h with constant rotation at 60 rpm. Following hybridisation, the solutions were removed and the arrays washed and stained on a fluidics station (Affymetrix FS450). Gene arrays were then scanned at an emission wavelength of 570 nm at 1.56 μm pixel-resolution using a confocal scanner (Affymetrix GeneChip Scanner 3000 7G). After scanning, the hybridisation intensity for each 25-mer feature was computed using Affymetrix GCOS version 1.3 software.

The raw data was then transferred into R/Bioconductor [77] and preprocessed using the Affy package [78], principally the RMA function, which relies on a robust multi-array averaging algorithm (RMA) for background adjustment, normalization, and summarization of the probe sets [79]. All probes mapping a CDS on P. falciparum genome (www.genedb.org, June 2007 update) were assigned in a probe set, giving a total of 5356 *Pf* CDS.

For a specific gene-analysis, we undertook a nonhierarchical clustering based on expression signals for each of the nine conditions (three cell lines: Δ*Pf*Sir2A, Δ*Pf*Sir2B, and wildtype (3D7) at three different time points: rings, trophozoites, and schizonts) for all the probe sets representing the genes of interest. The genes were clustered by measuring similarity of shapes using Pearson coefficients. The unsupervised clustering program enabled us to discriminate biological clusters (ArrayMiner, OptimalDesign). ArrayMiner software searches for solutions of minimal total variance in a given number of clusters. However, unlike most other currently available methods, ArrayMiner does not rely on k-means in its search for high quality clusters as it uses a GGA optimisation process (the total variance of the clusters being minimized). To draw heatmaps, the expression values for each gene were slid vertically so that their average was zero (zero mean normalisation) then multiplied so that the length of the associated vector (square root of the sum of squares of all values) be equal to 1 (unit-norm normalisation of length 1). The resulting contrasts were plotted on a scale from −3 (blue, low expression) to +3 (yellow, high expression).

### CSA selection of P. falciparum-infected erythrocytes.

Parasites were cultured to trophozoite stage and enriched away from uninfected erythrocytes by gelatin selection [80] and bound to CSA as described previously [81]. Parasite lines were selected in parallel five times, which was enough to change R1945 binding profile of 3D7 to close to 100% positive suggesting that selection had gone to completion.

### FACS analysis.

Expression of Var2CSA-specific PfEMP1 was assessed by FACS using established methods [47]. In short, cells were sequentially incubated with R1945 (rabbit serum), and Alexa-Fluor 488 conjugated donkey anti-rabbit Ig (Molecular Probes)/ethidium bromide (BioRad). All incubations were performed in PBS/0.1% casein for 30 min at room temperature. Samples were analysed on a Becton-Dickinson FACSCalibur flow cytometer. Using FlowJo (TriStar) IgG binding for each sample was expressed as the geometric mean fluorescence intensity (MFI) for infected erythrocytes (ethidium bromide positive), after subtracting the MFI for uninfected erythrocytes.

### qPCR.

For the expression analysis RNA was harvested from ring-stage parasites synchronised in their previous erythrocytic cycle by treatment with sorbitol. Cooled saponin lysed parasites were resuspended in TRIzol and RNA harvested as per manufacturers recommendations (Invitrogen). RNA was further purified using RNeasy columns (Qiagen) and treated with Turbo DNase to remove any residual DNA (Ambion). RNA samples were tested for gDNA contamination by PCR. First strand cDNA synthesis took place using a Retroscript kit (Ambion) or Superscript III Reverse Transcriptase III (Invitrogen). Extraction of gDNA was performed at trophozoite stage. qPCR primers used for analysing expression of the 3D7 *var* repertoire was repeated as previously described [46]. cDNA quantification was performed on a Roche LC480 light cycler. Transcript quantification was performed by comparing to standard dilution series of gDNA for all *var* genes and then normalising for cDNA concentration with the expression level of actin (PFL2215w). In experiments describing the expression of pUpsB/C plasmids *hDHFR* was normalised against *Msp8* (another ring-stage expressed gene) and *hsp86* was used to normalise *bsd* expression (*bsd* is driven by the *hsp86* promoter).

Plasmid copy number was quantified using the level of *bsd* versus the signal from the single copy gene *hsp86*. *hdhfr* transcript levels were normalised against the expression of the ring-expressed *msp8* gene and then divided by the plasmid copy number calculated previously. ΔPfSir2 parasites also contain hDHFR as a selectable marker for gene disruption therefore the *hdhfr* transcript level from these parasites without pUpsB/C was subtracted from parasites harbouring these plasmids as to normalise for the *hdhfr* signal from the UpsB-driven *hdhfr* alone. *bsd* transcript levels were normalised against the expression of *hsp86* and further by taking into account plasmid copy number. Standard error was calculated across the three biological replicates and used to derive confidence intervals. All expression values were normalised against wildtype parasites with an active UpsB/C-*hdhfr* gene as a measure of the suppression status of all other samples.

## Supporting Information

Figure S1 Click here for additional data file. Alignment of the Catalytic Domain of Sir2A and B Paralogues with Other Sirtuins
Shading level denotes level of conservation within aligned sequences. Boxes 1, 2, and 3 form the “Rossman fold” and are the binding pocket for NAD^+^ . Residues that are considered critical in Hst2 for the catalytic activity of the NAD^+^ binding pocket are marked with “*” and these are completely conserved in PfSir2B. “#” marks the cystine residues that form a triad for binding zinc ions and these are completely conserved in PfSir2B. Boxes 4 and 5 denote the region that binds the acetylated substrate and this shows diversity between PfSir2A and PfSir2B and other sirtuin members. “*” residues highlighted in boxes 4 and 5 are those that make contact with the acetylated substrate in Hst2 and these also show differences. Hs, Homo sapiens; Pf, P. falciparum; Pv, P. vivax; Sc, Saccharomyces cerevisiae.
(1.11 MB TIF)

Figure S2 Click here for additional data file. Genomic Characterisation of PfSir2 Mutants
(A) Pulse field gel and southern blot using plasmid backbone as a probe. Membrane shows integration of plasmid into two high molecule weight chromosomes consistent with Chromosomes 13 and 14 and proves the identity of these parasites for further analysis.
(B) Rep20 fingerprinting of parasite lines signifying that they are all 3D7. *Var* genes and subtelomeric ends are highly polymorphic between strains and thus Rep20 fingerprinting provides evidence that comparisons of *var* (and other genes) within subtelomeric regions is comparable between all these cell lines. Arrow points to an RFLP between ΔPfSir2A and 3D7.
(905 KB TIF)

Figure S3 Click here for additional data file. Physical Map of Subtelomeric Gene Expression in Sir2-Disrupted Lines
Physical map of subtelomeric and colour-coded expression of each gene denotes expression level and Sir2-disrupted line it is expressed in. Subtelomeric ends of each of the 14 P. falciparum chromosomes are accompanied by graphs of the expression of subtelomeric genes. Expression profile of every biological replicate for each individual gene across the three intraerythrocytic time points is shown as a dot while the average expression across the replicates is shown as a line: red, ΔPfSir2A; blue, ΔPfSir2B; and grey, wildtype 3D7 parasites.
(3.73 MB PDF)

Figure S4 Click here for additional data file. Raw Expression Values of *var* Genes in 3D7 and Sir2-Disrupted Parasites
*Var* genes were grouped according to the conserved promoter type that drives their expression and plotted on a graph as a log_2_ values. Expression values of *var* genes in 3D7 are in green, ΔPfSir2A in red, and ΔPfSir2B in blue. Highly statistically different values from 3D7 are marked with a “*.” As seen with Gaussian self-clustering disruption of PfSir2A has a greater effect on the derepression of UpsA and UpsC-driven *var* genes suggesting a role for this molecule in their suppression, whereas UpsB *var* gene suppression is controlled by PfSir2B. This Affymetrix expression data is corroborated by qPCR expression values outlined in Table S2.
(576 KB TIF)

Figure S5 Click here for additional data file. Analysis of *var* Genes by qPCR on CSA Selected Parasite Lines
In standard culture conditions 3D7 parasites express a standard set of *var* genes and largely do not express *var2CSA*. Upon selection of infected red blood cells to bind to CSA parasites expressing *var2CSA* are highly enriched and as a consequence of mutual exclusion the expression of all other *var* genes in these parasites are suppressed as they drop to near basal levels.
(501 KB TIF)

Table S1 Click here for additional data file. Log_2_ Ratio of All Genes Differentially Expressed at Any Stages in ΔSir2A and ΔSir2B Parasite Lines
(54 KB XLS)

Table S2 Click here for additional data file. Expression of 3D7 *var* Repertoire in PfSir2A, PfSir2B, and Wildtype Parasites before and after Selection on CSA
(68 KB XLS)

Text S1 Supplementary Information (52 KB DOC)

## Abbreviations

bsd: blasticidin-S deaminase
CSA: chondroitin sulphate A
FACS: flow cytometry
PfEMP1: Plasmodium falciparum erythrocyte membrane protein 1
PfSir2A: P. falciparum silent information regulator A
PfSir2B: P. falciparum silent information regulator B
qPCR: quantitative PCR
sir: silencing information regulator
sir2: silent information regulator 2
