# Supplementary figures and images for: Sir2 Paralogues Cooperate to Regulate Virulence Genes and Antigenic Variation in Plasmodium falciparum
Source: PLoS Biol. 2009 Apr 14;7(4):e1000084. doi: 10.1371/journal.pbio.1000084 (PMC2672602; doi:10.1371/journal.pbio.1000084)

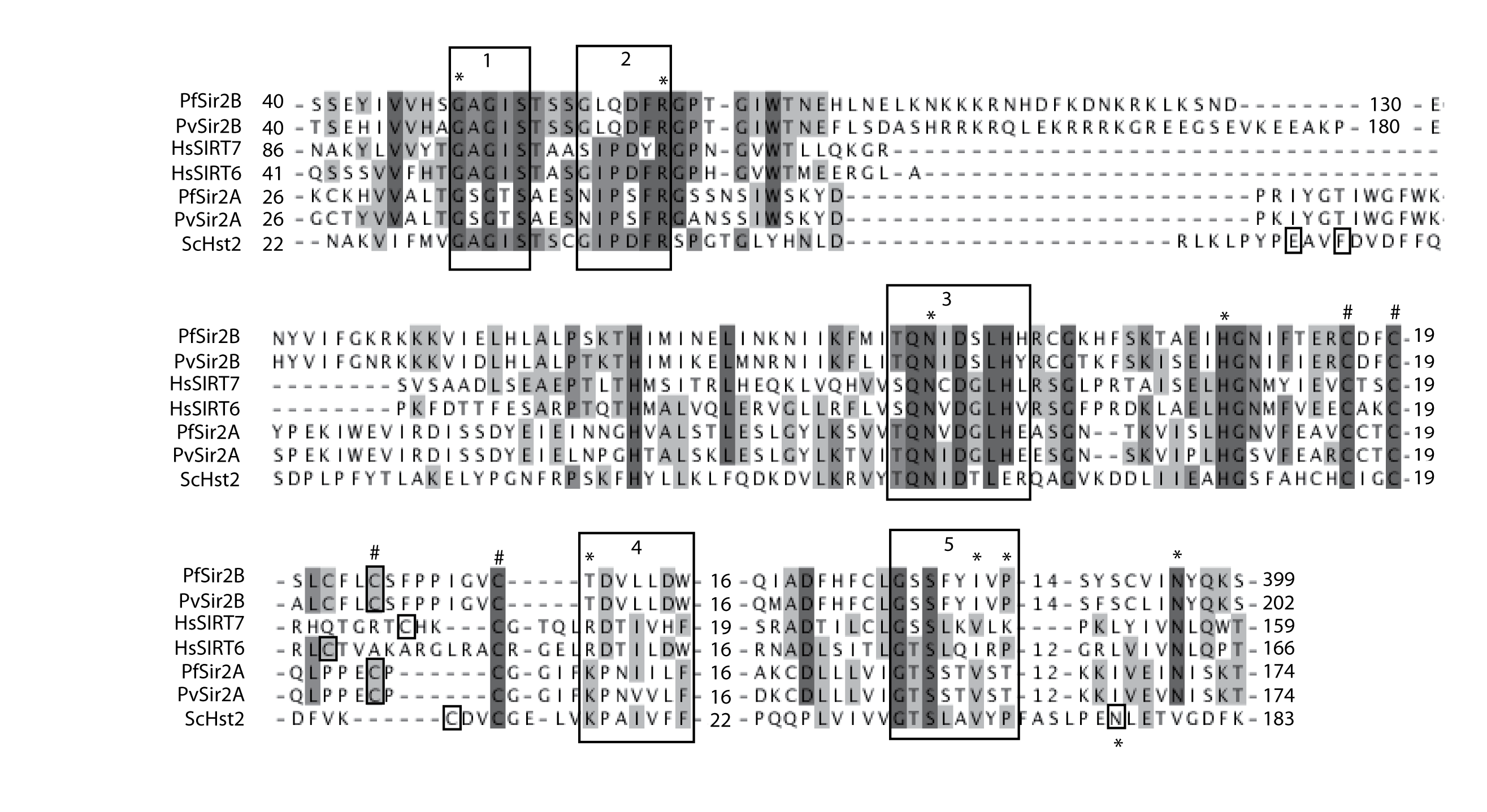

Supplement: Figure S1 [file pbio.1000084.sg001.tif]

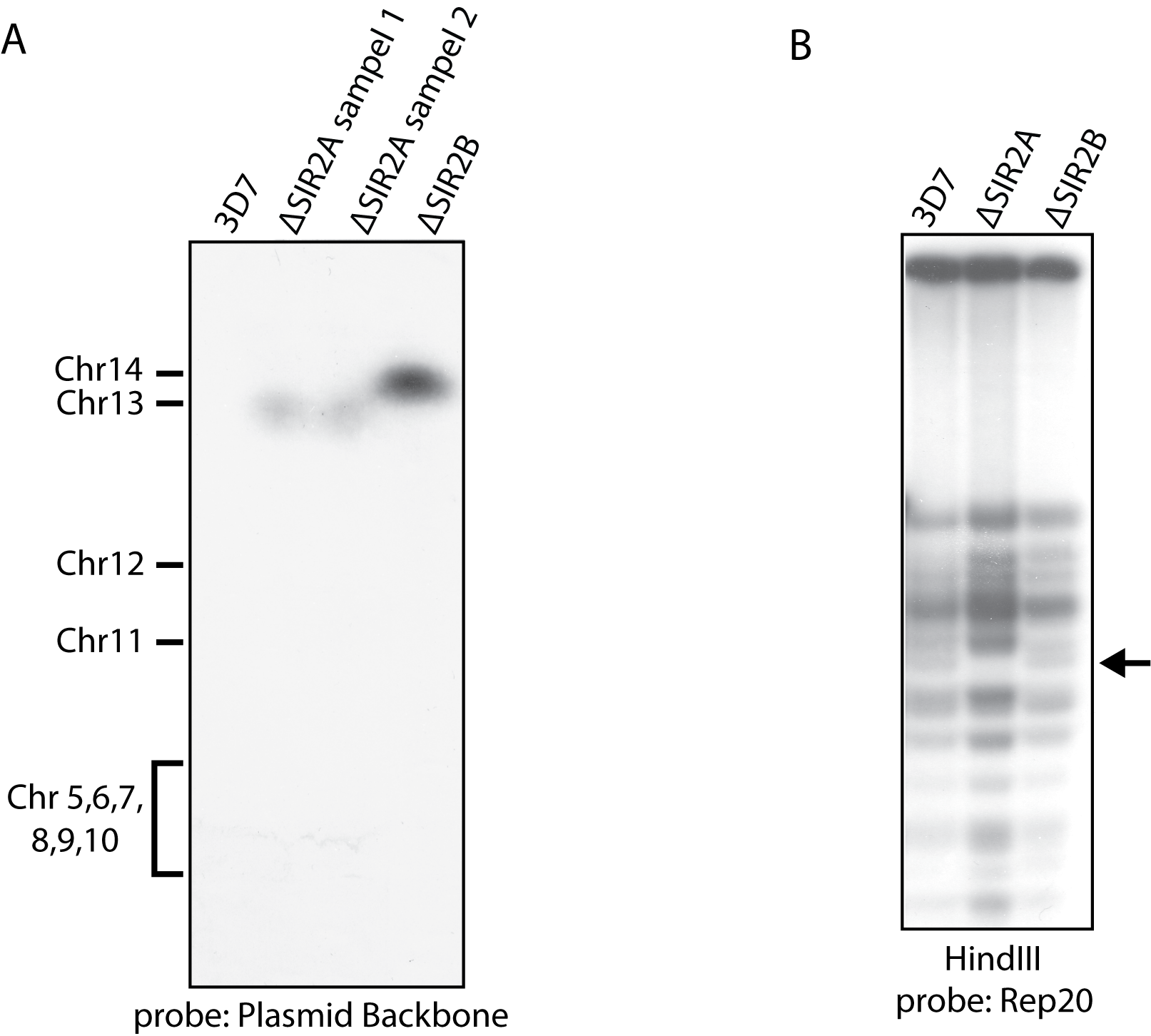

Supplement: Figure S2 [file pbio.1000084.sg002.tif]

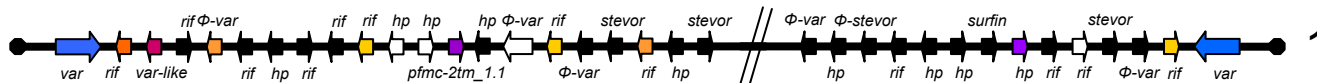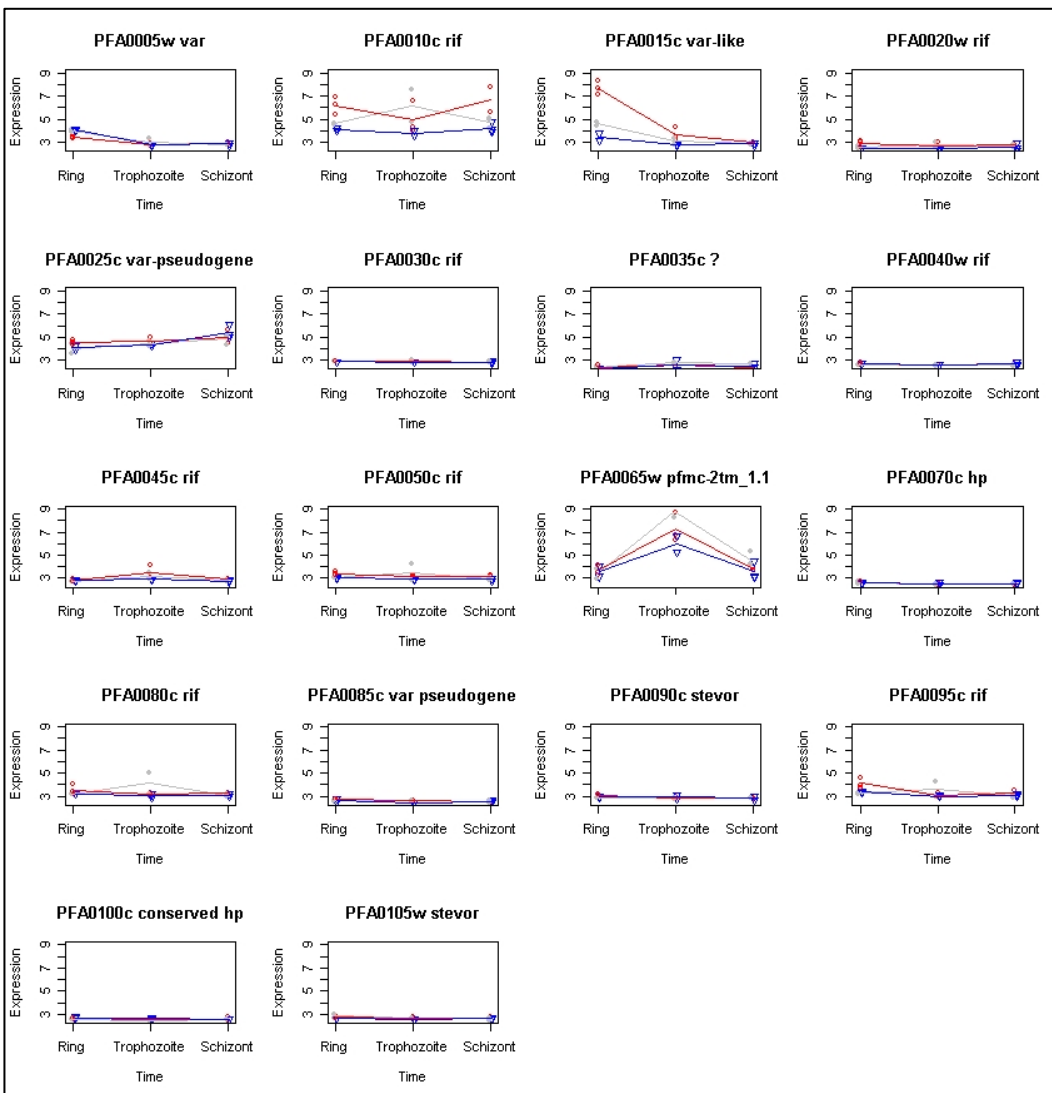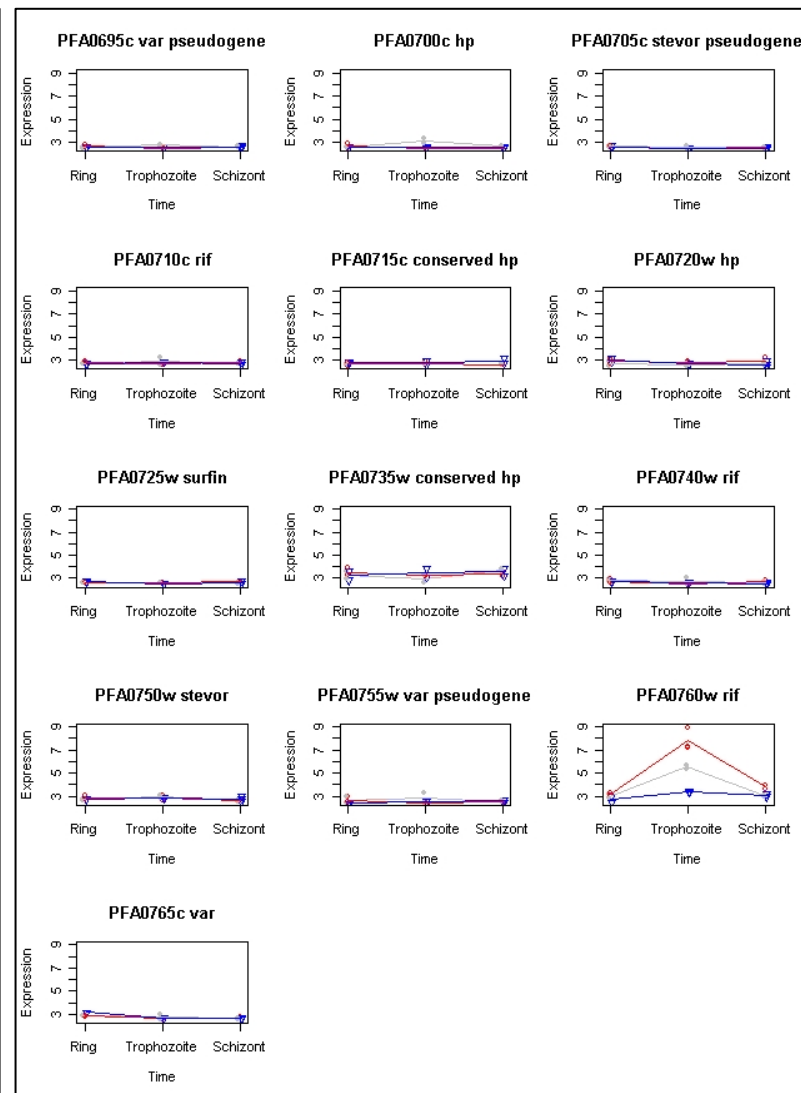

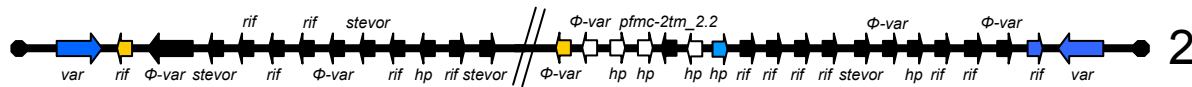

2

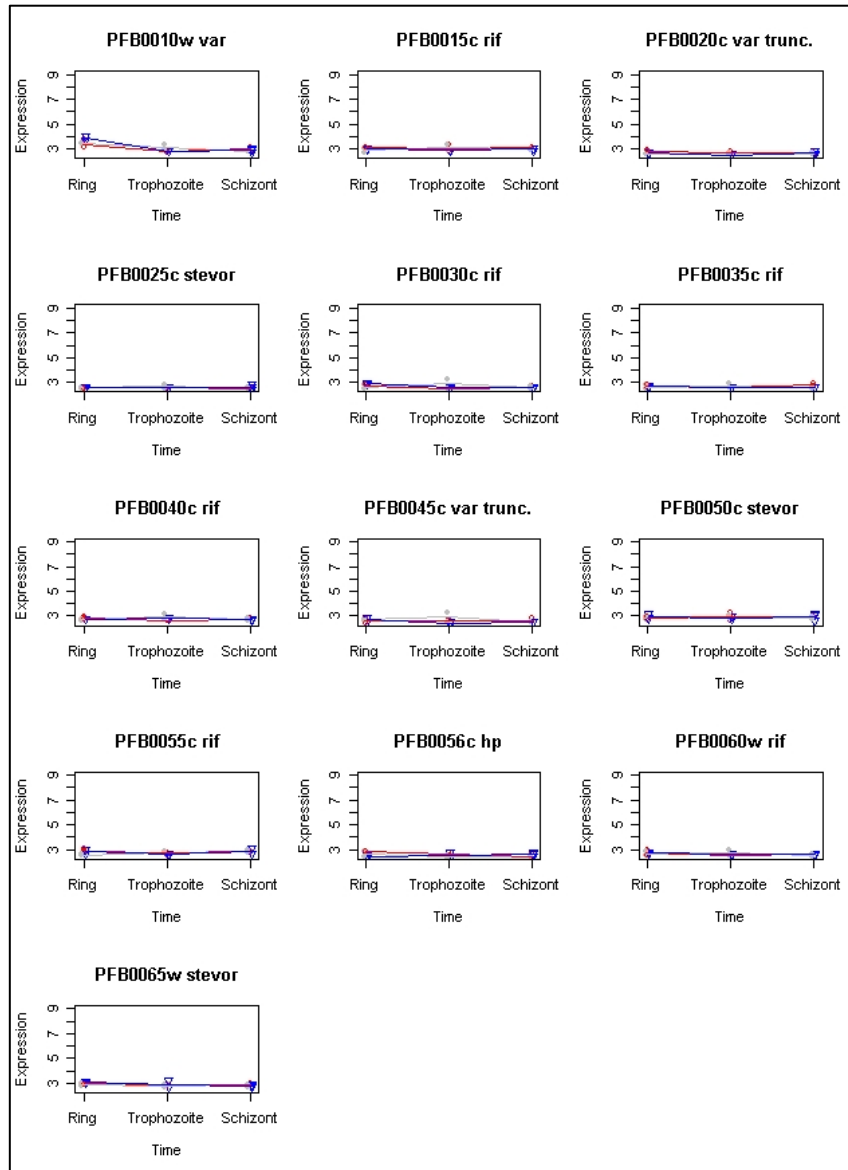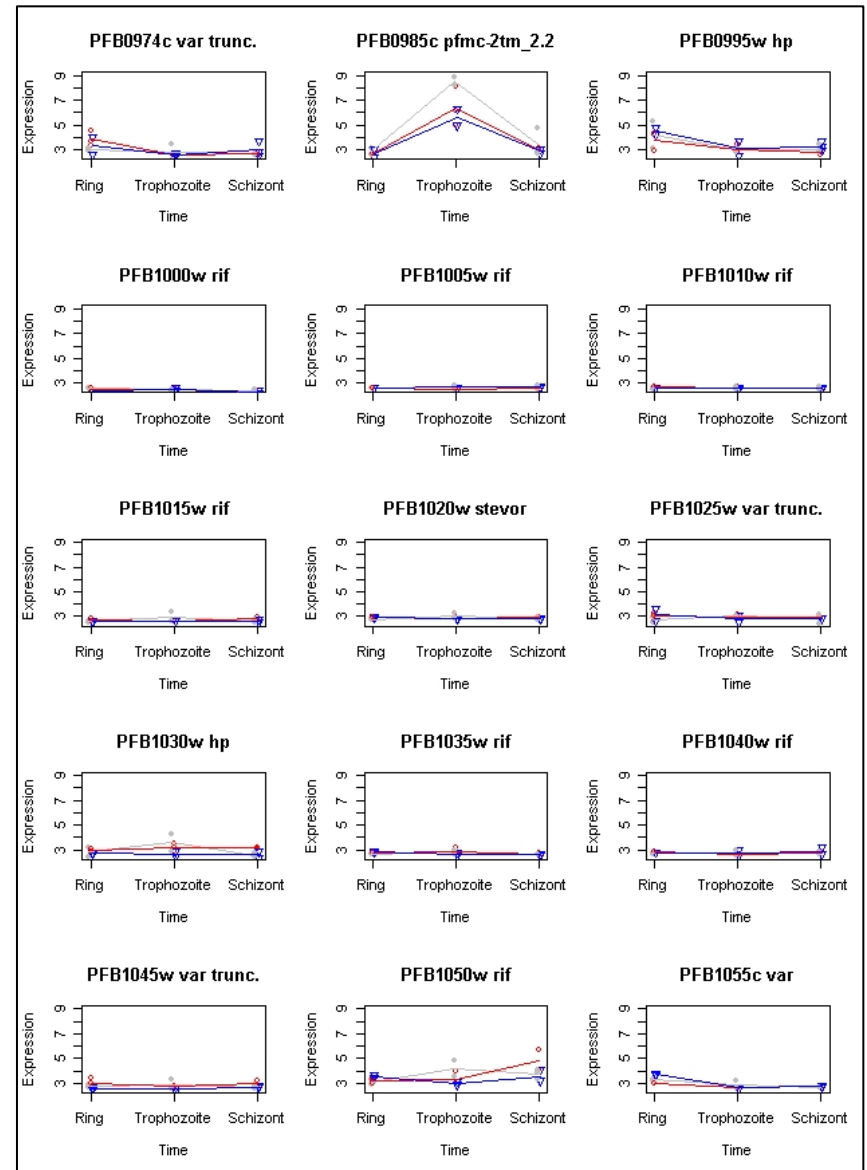

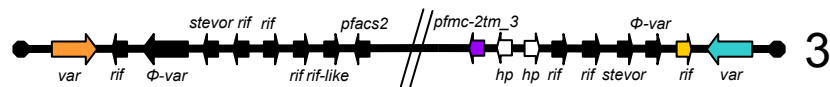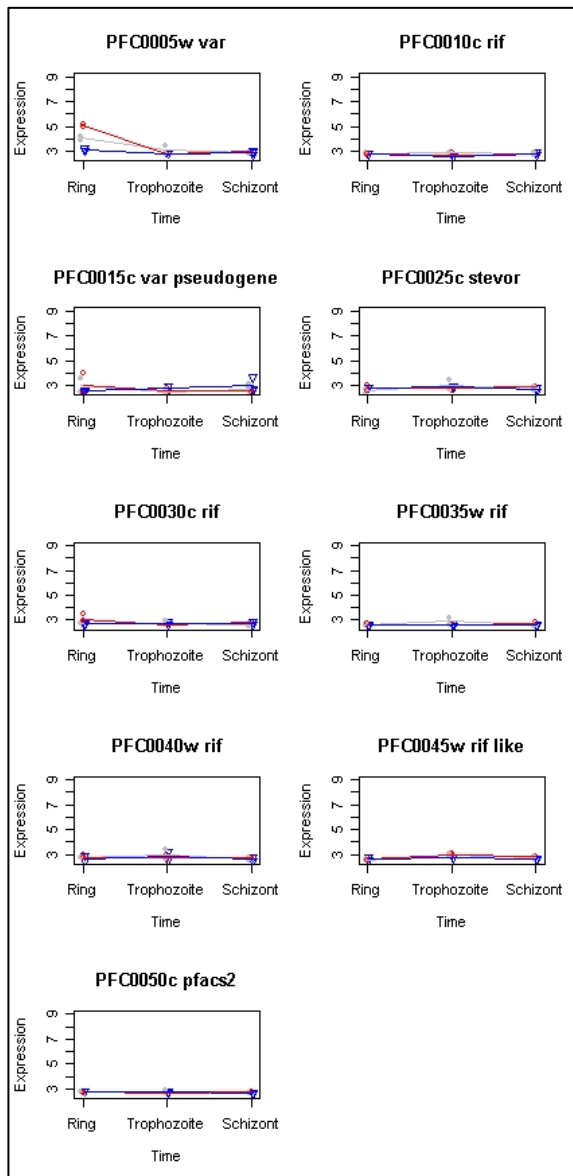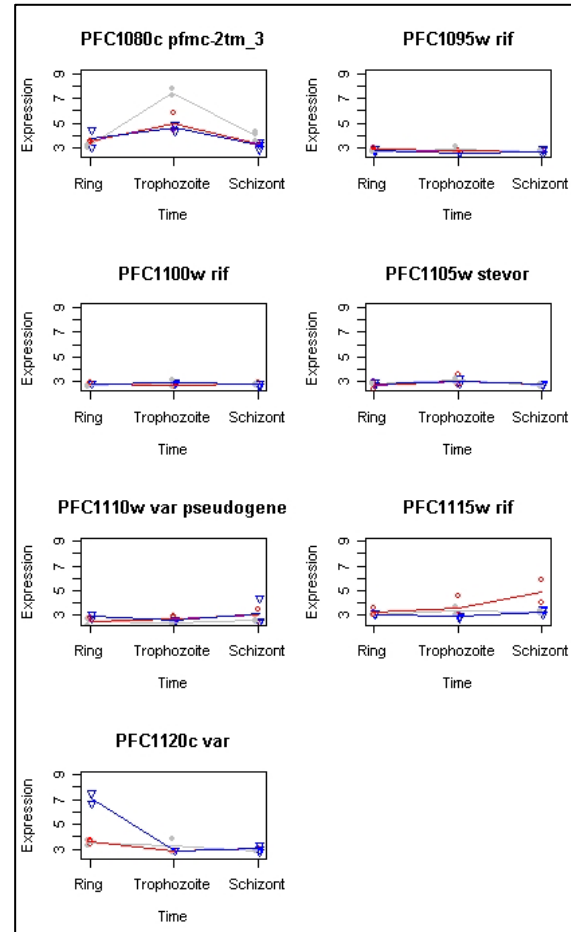

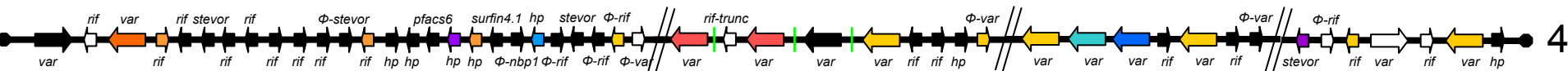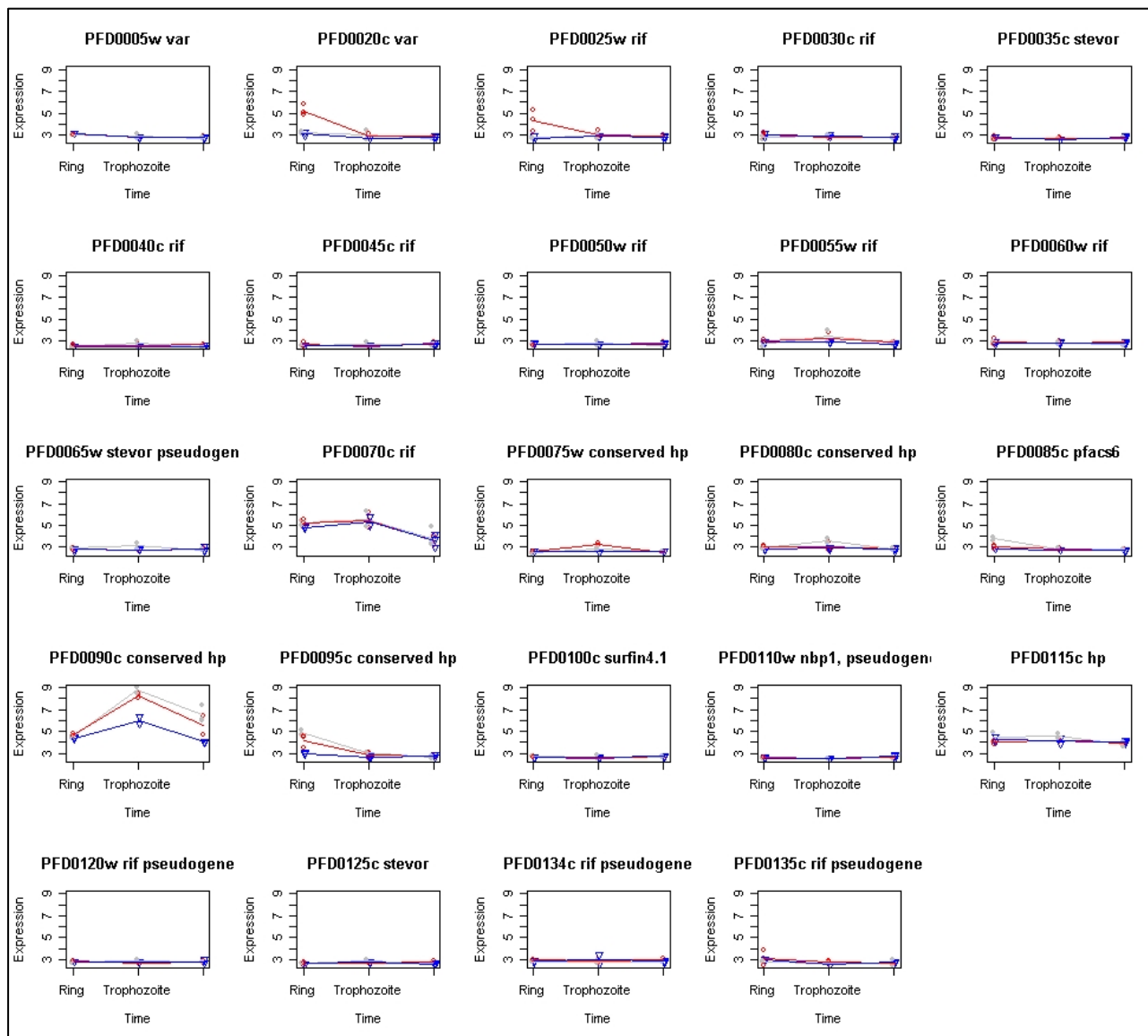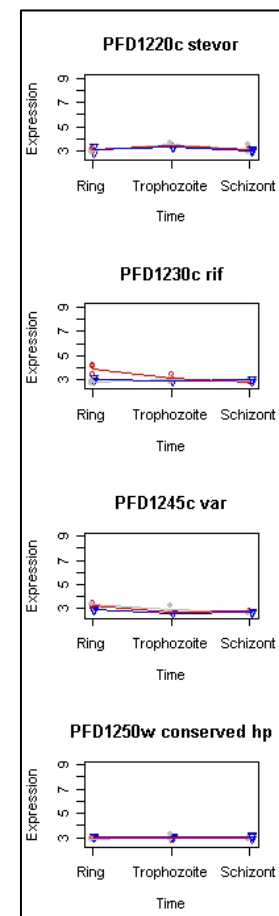

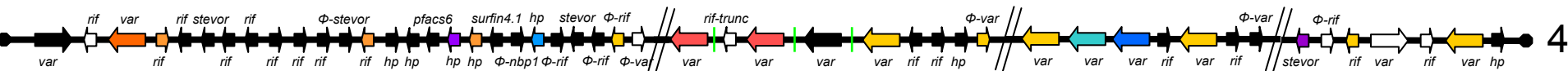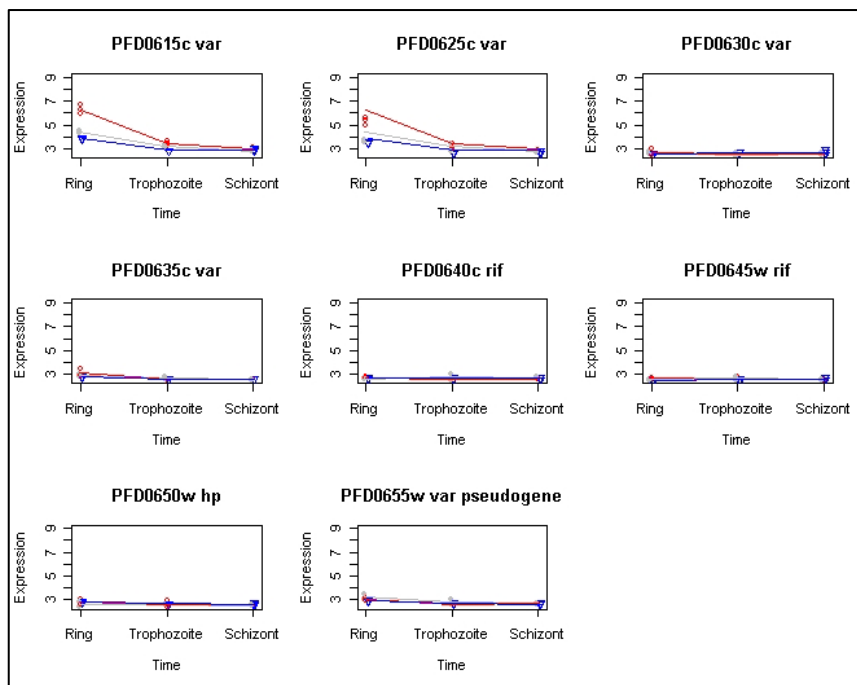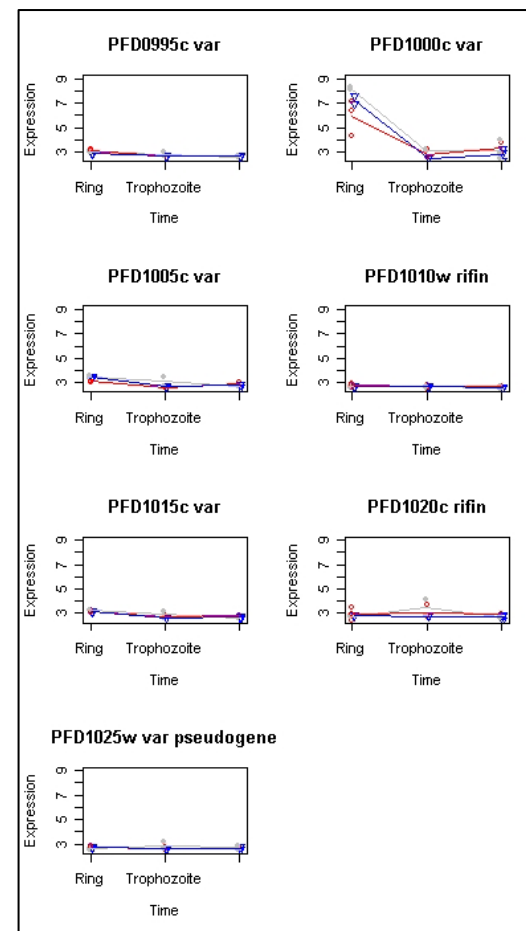

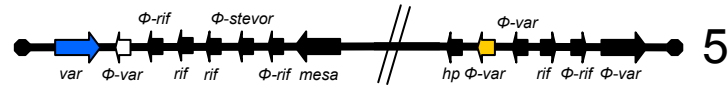

5

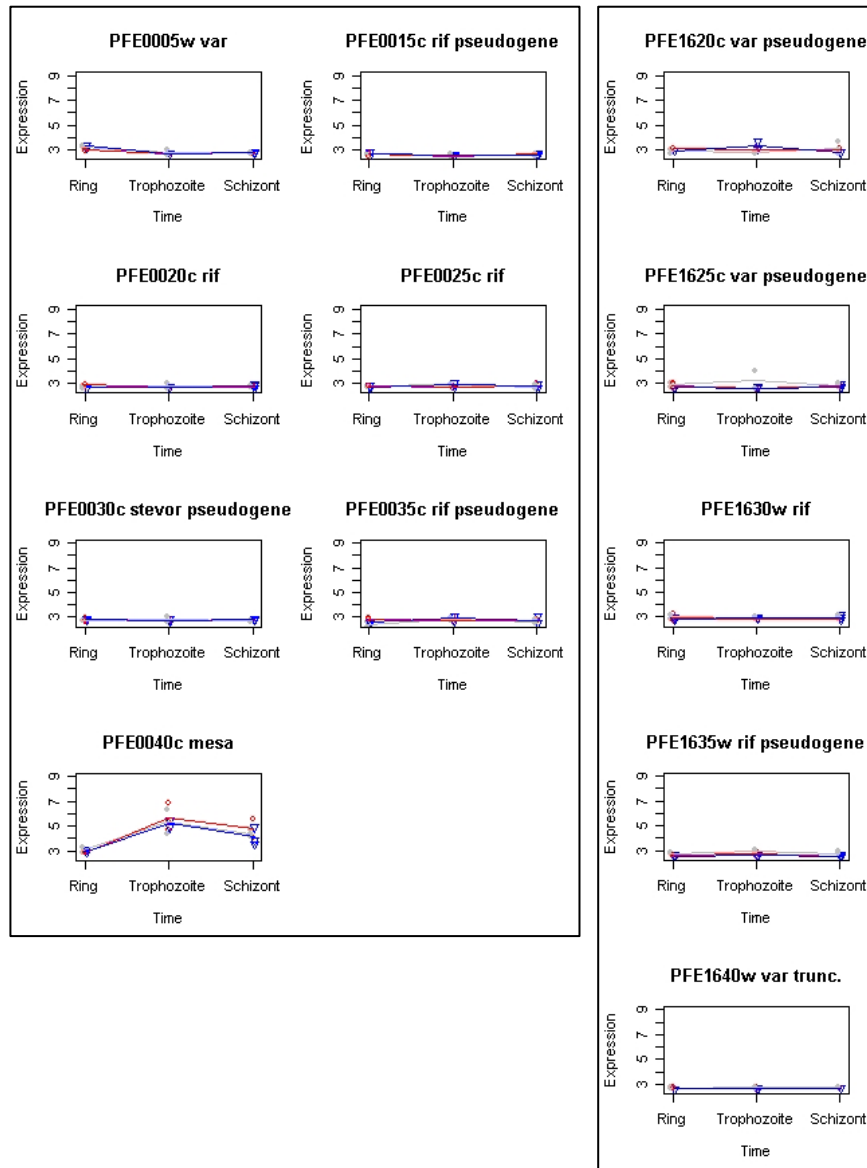

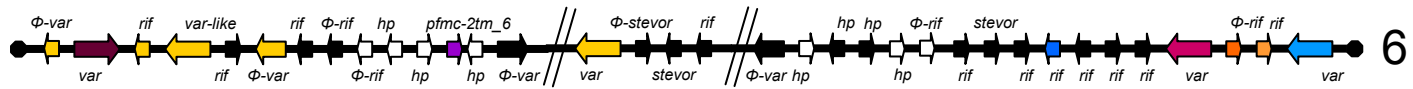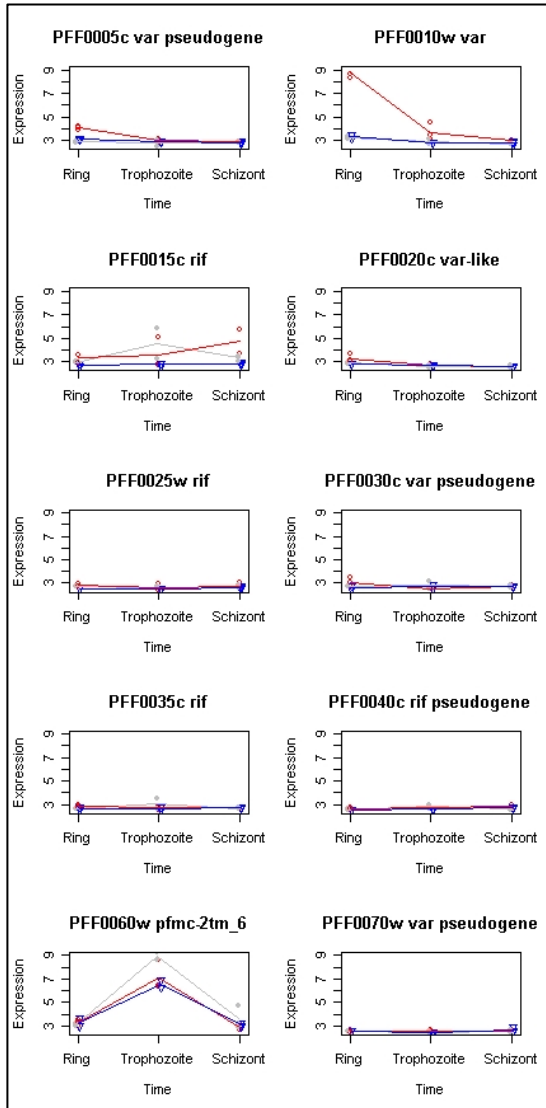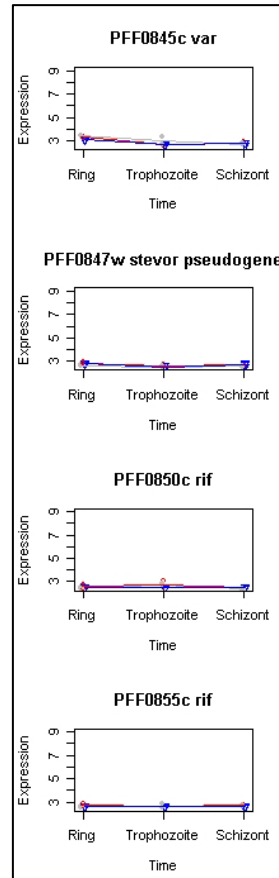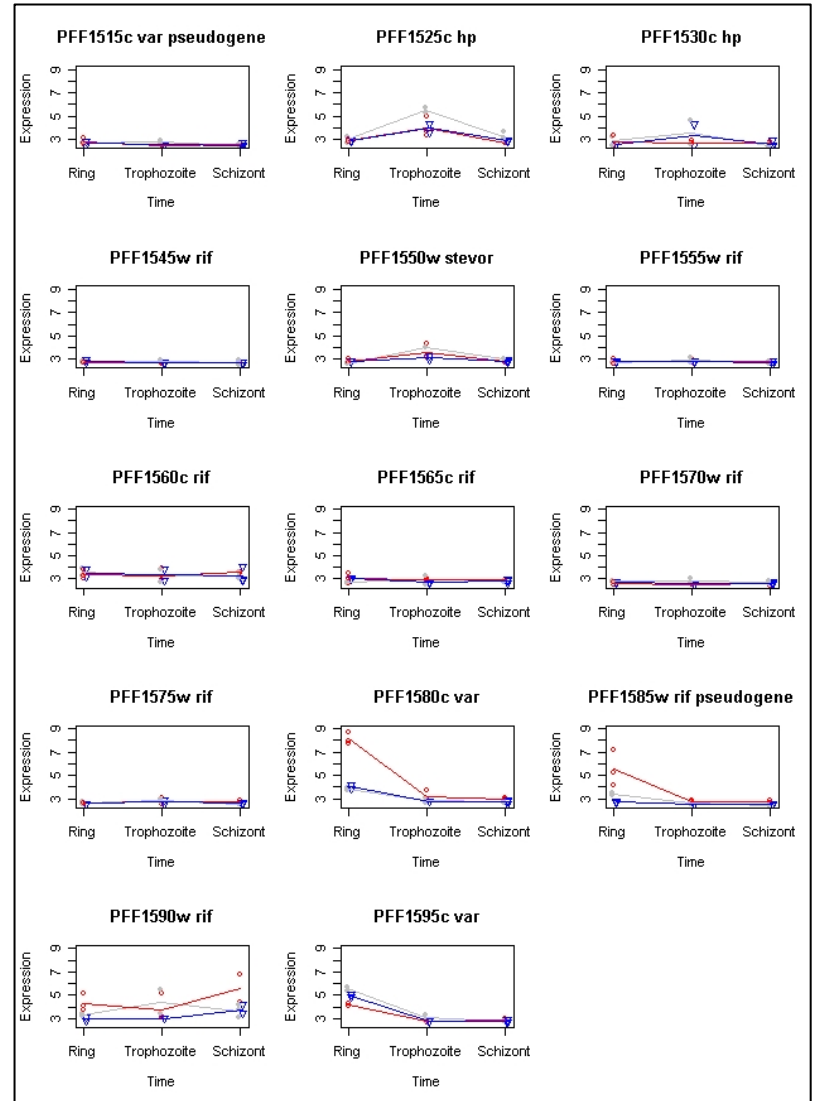

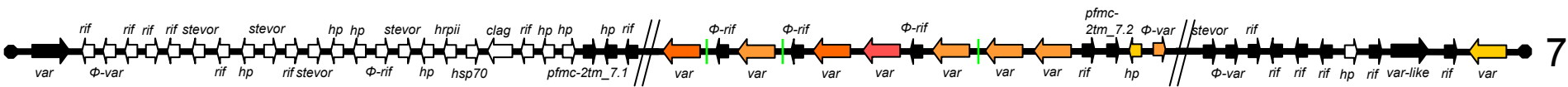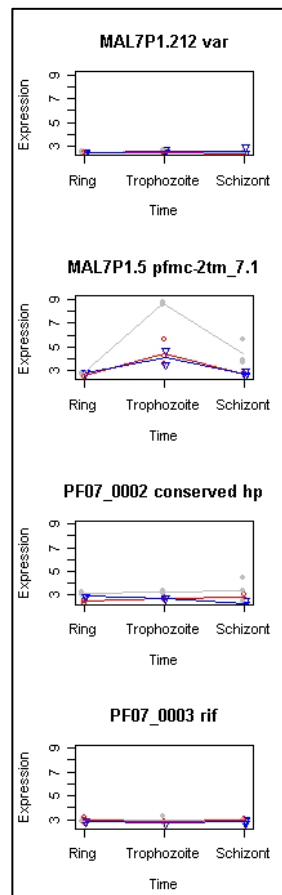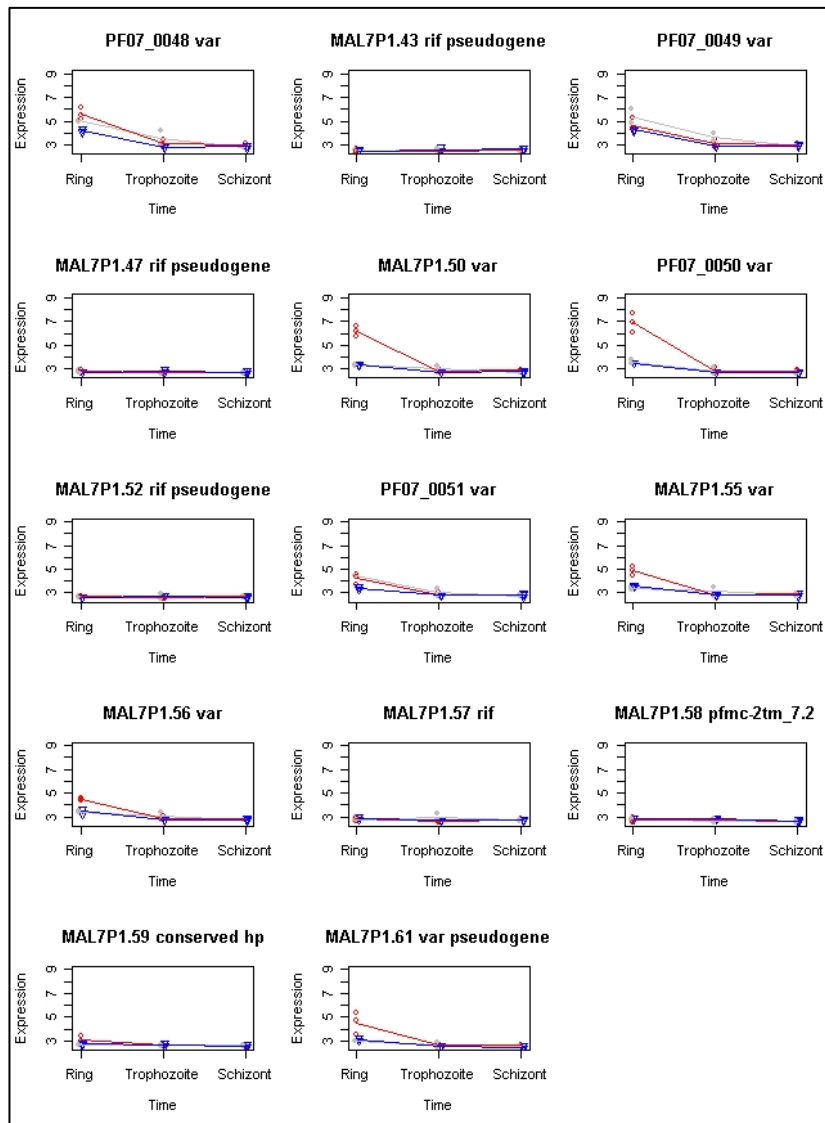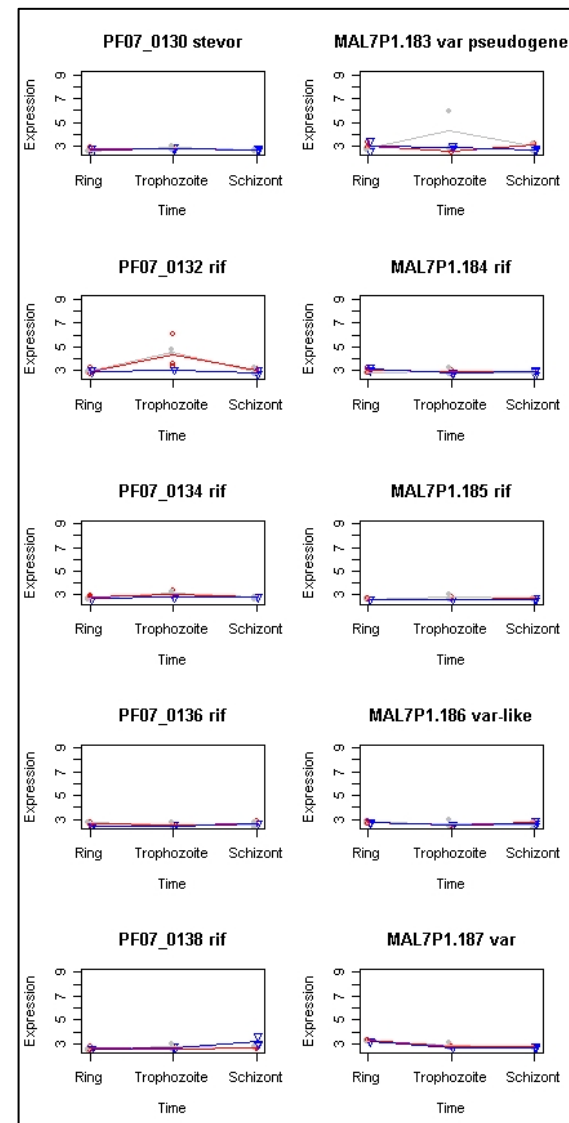

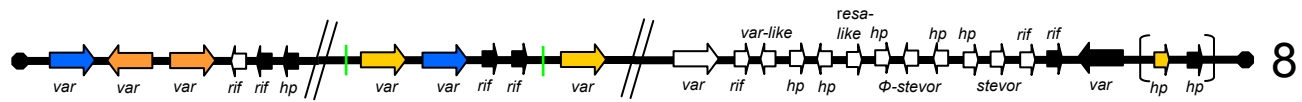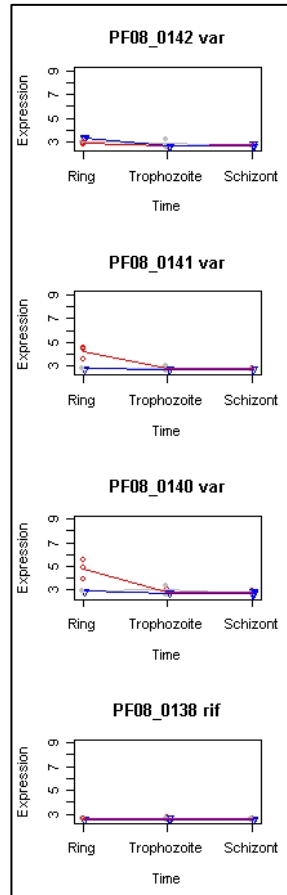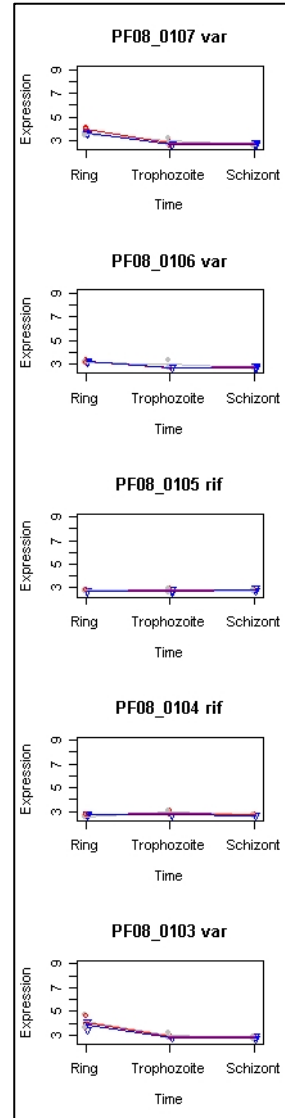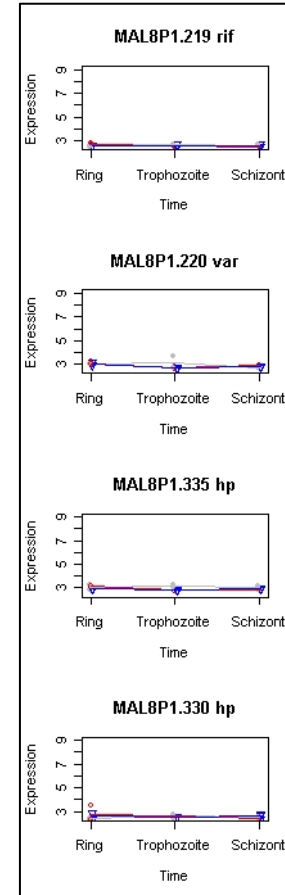

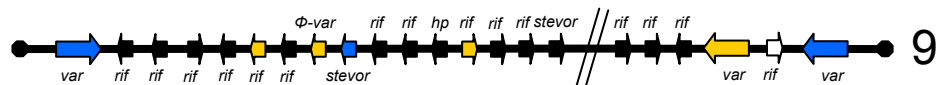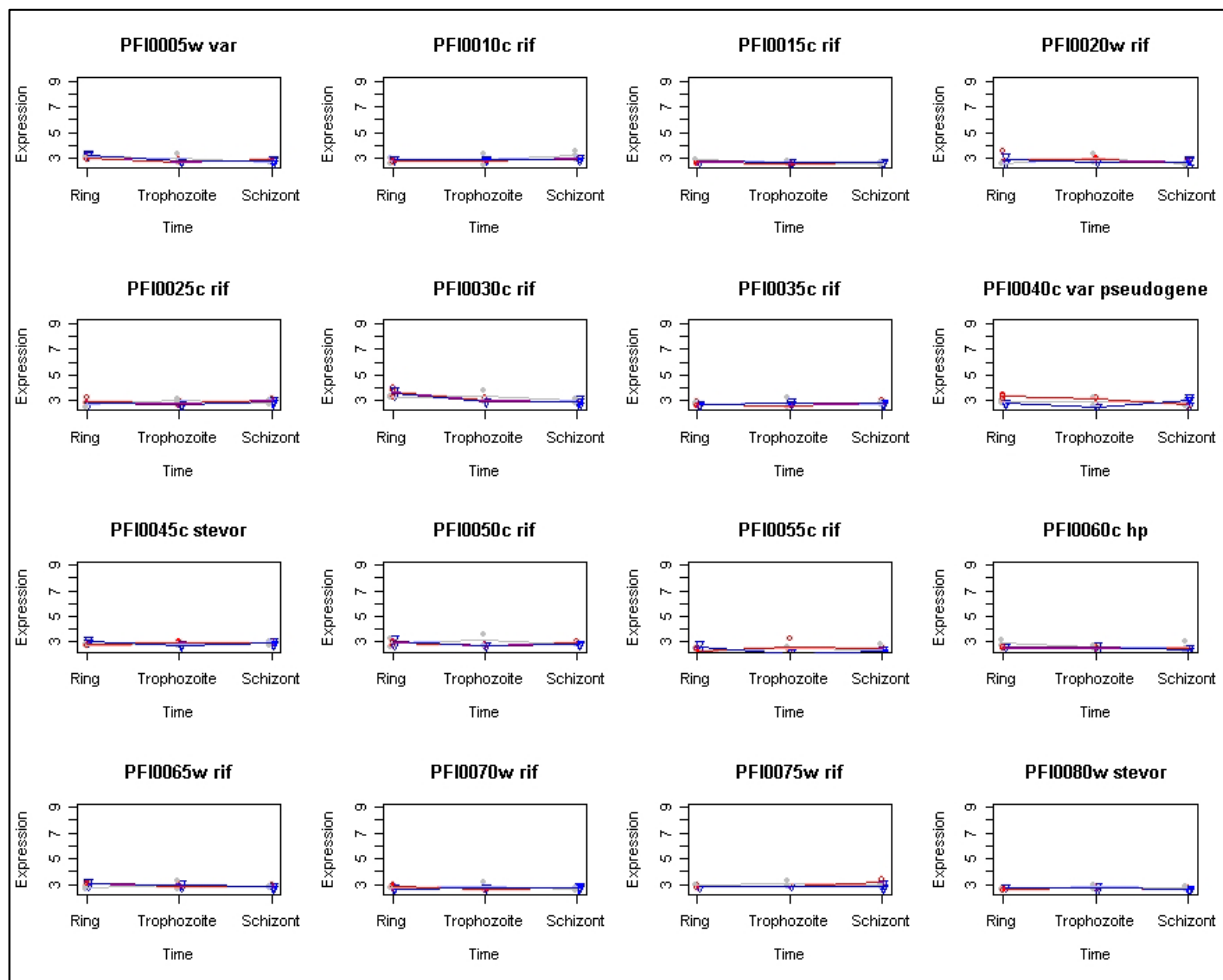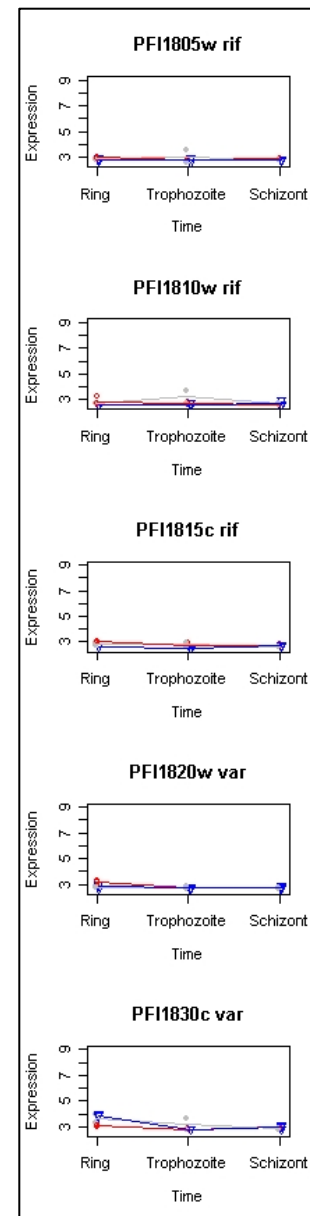

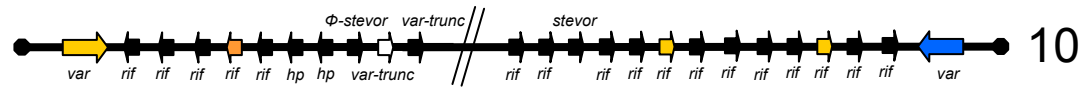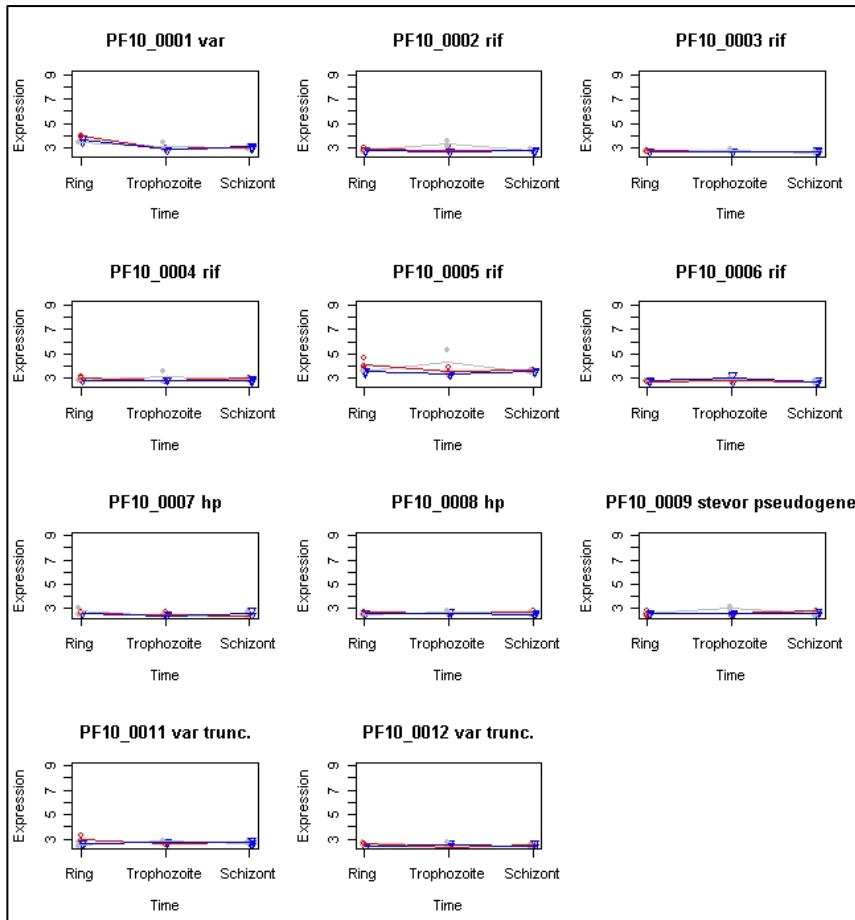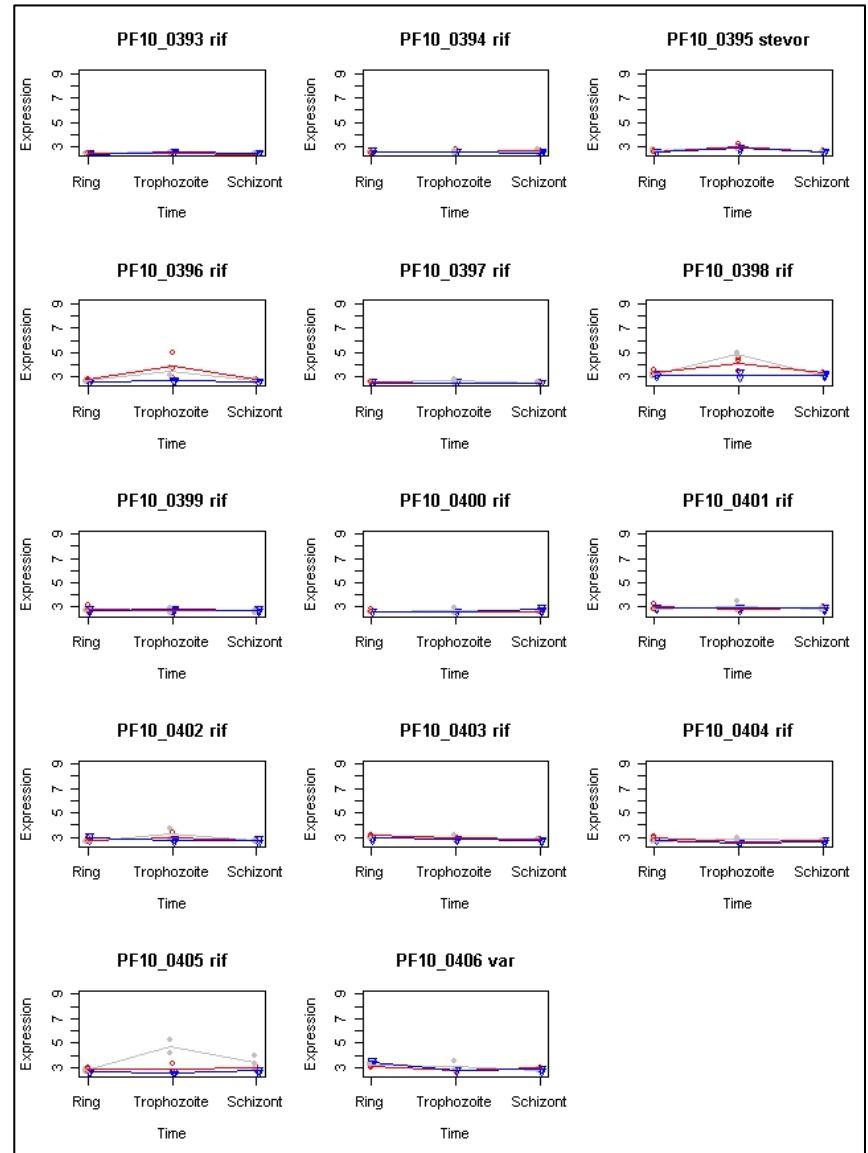

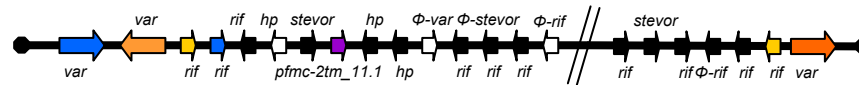

11

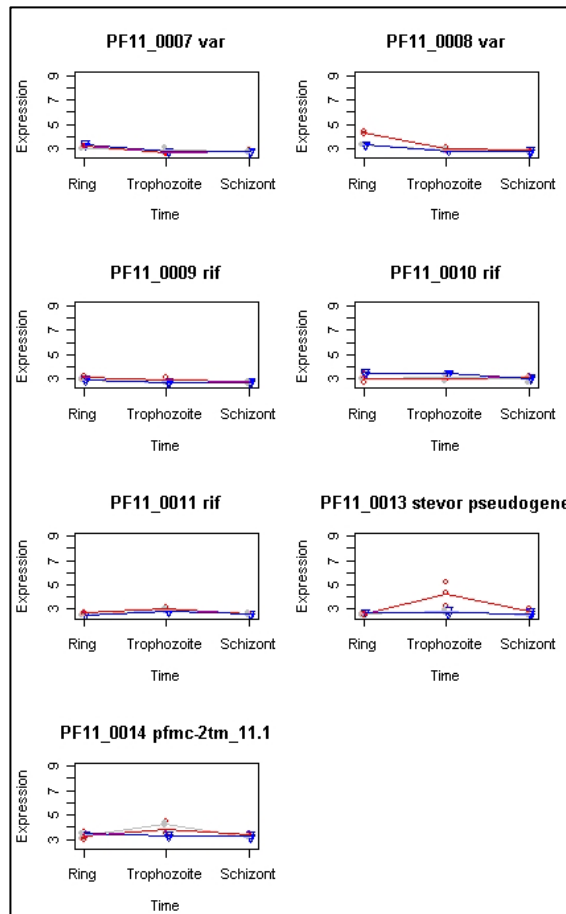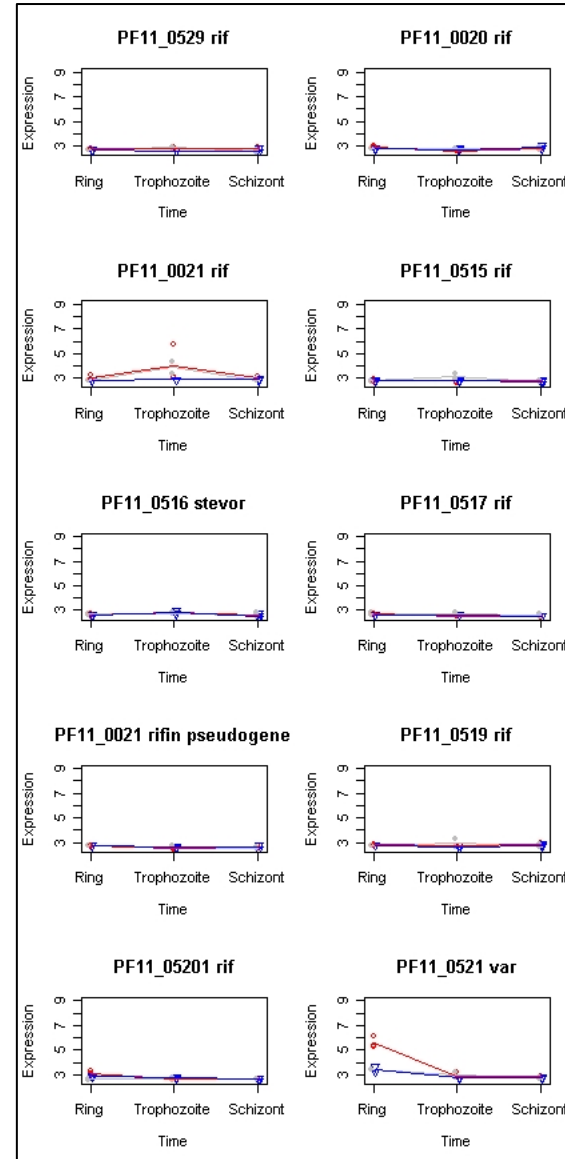

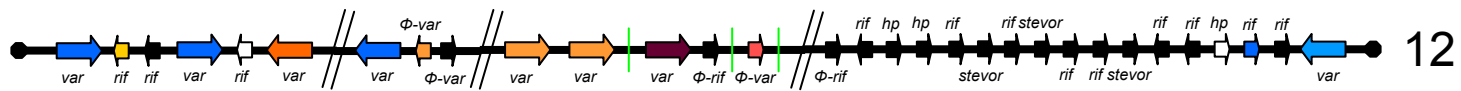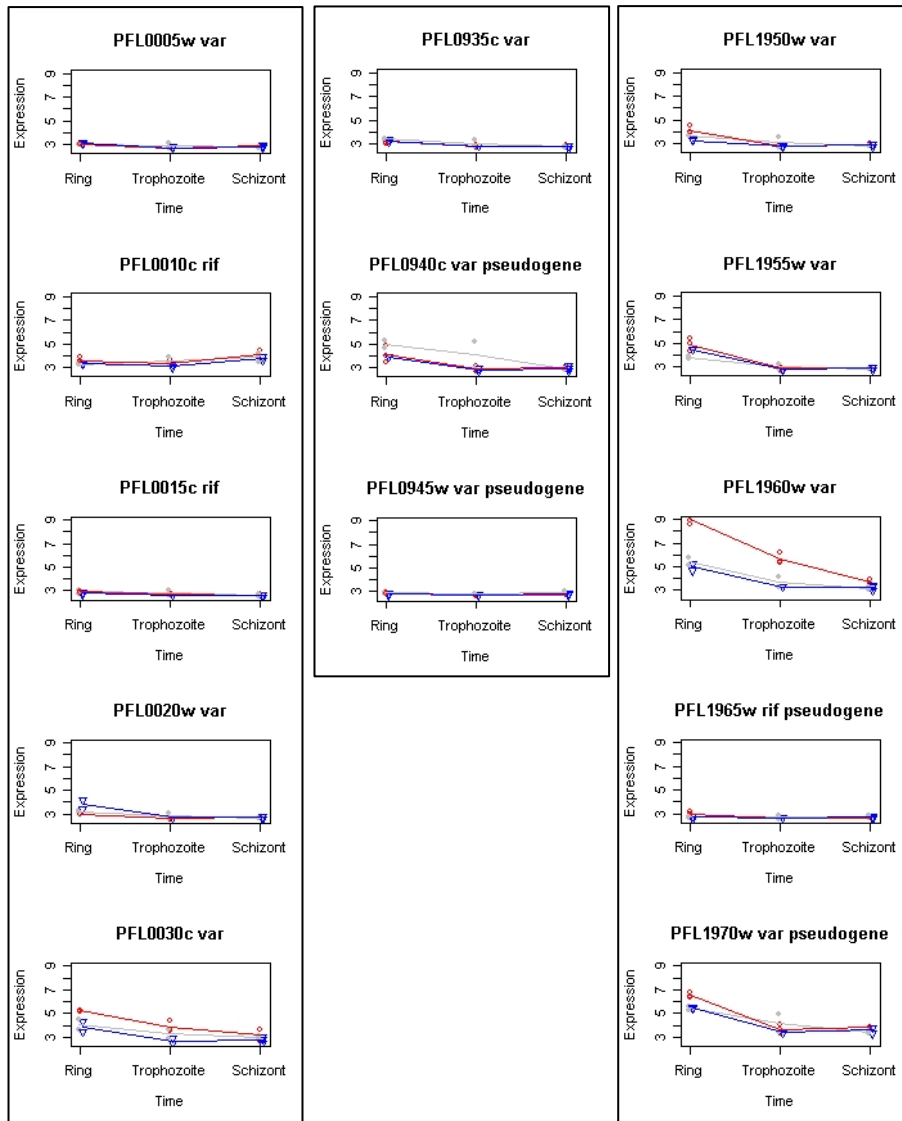

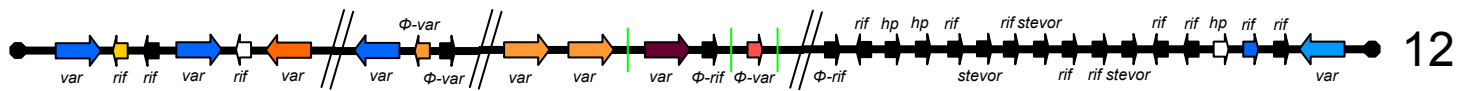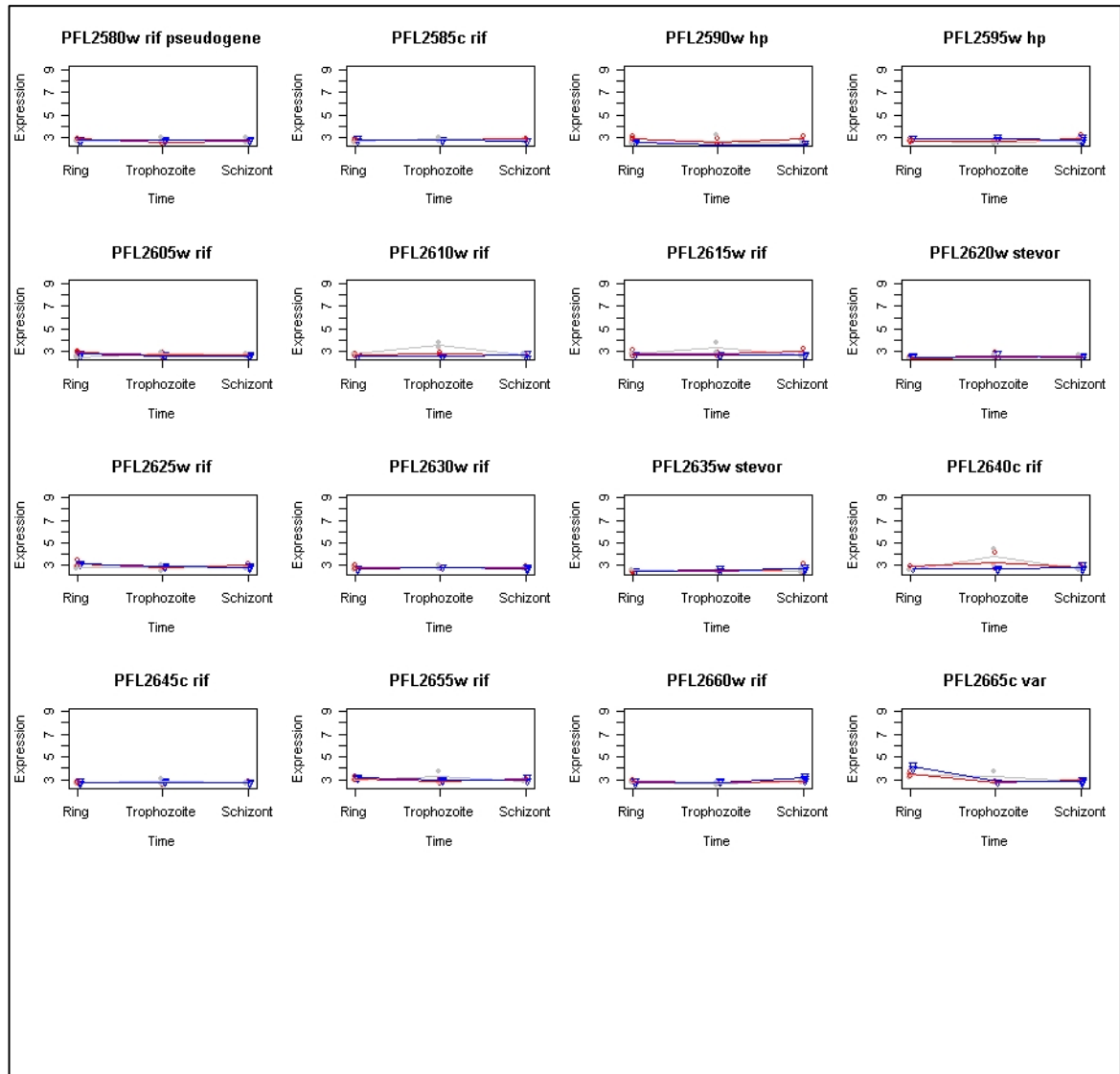

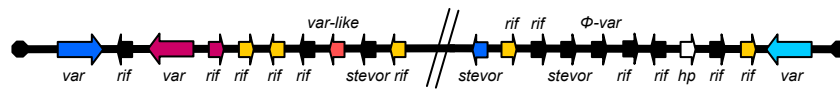

13

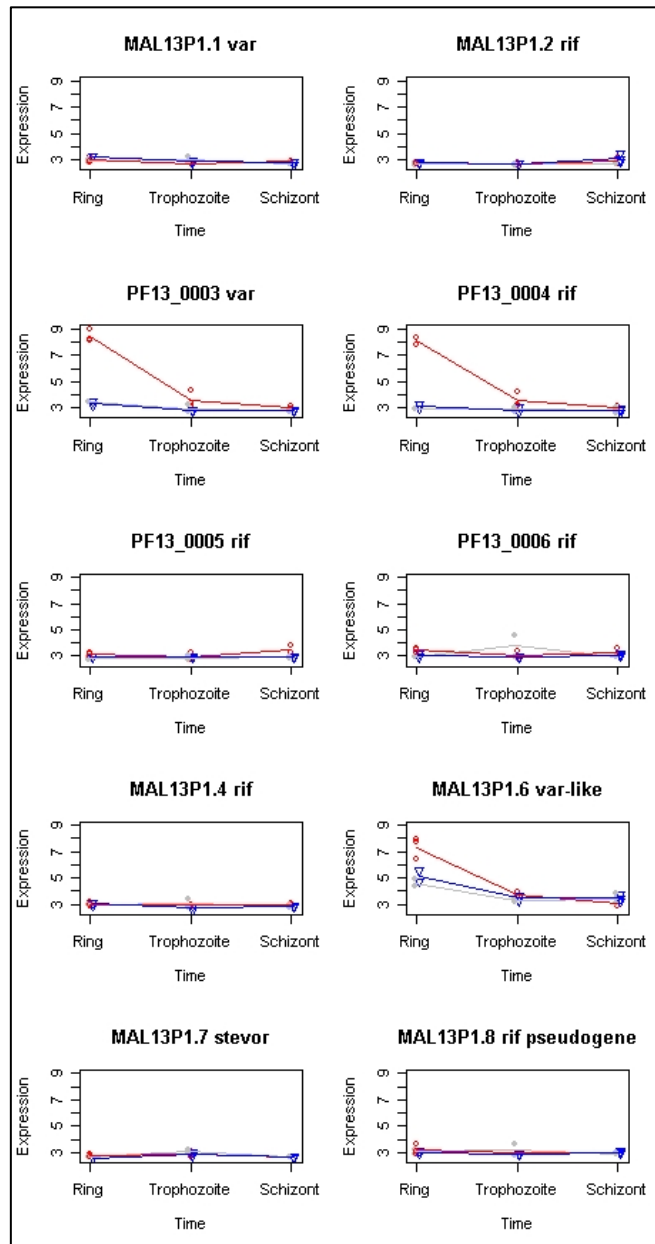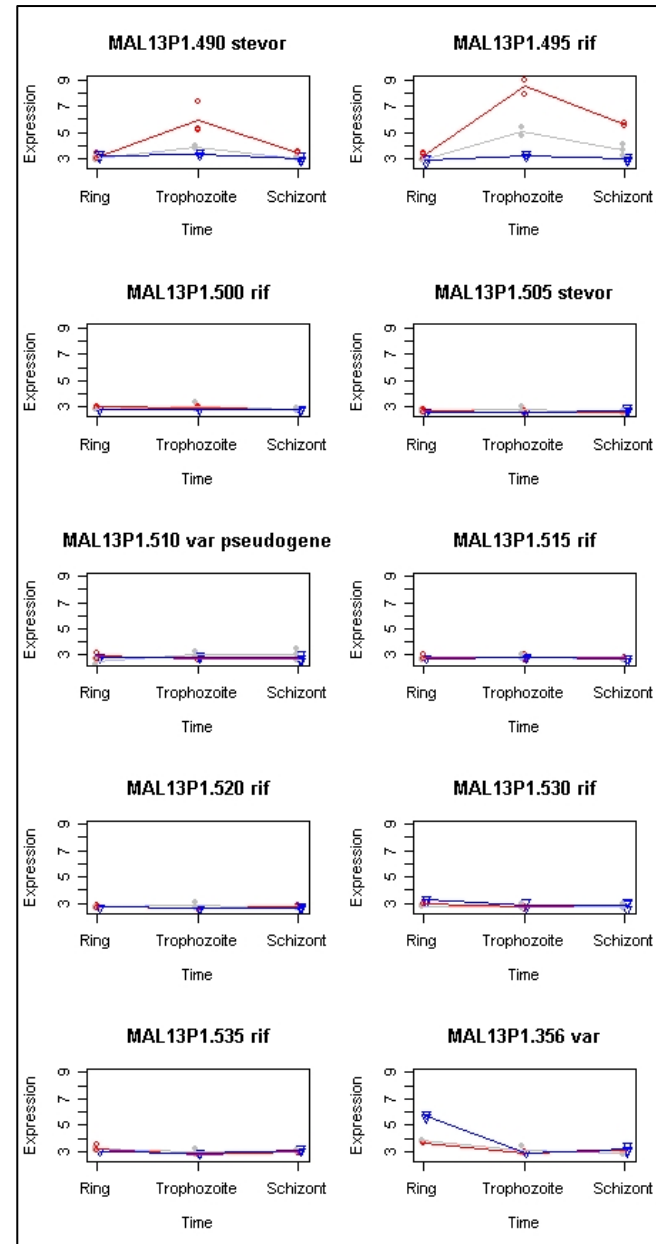

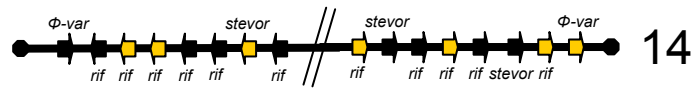

14

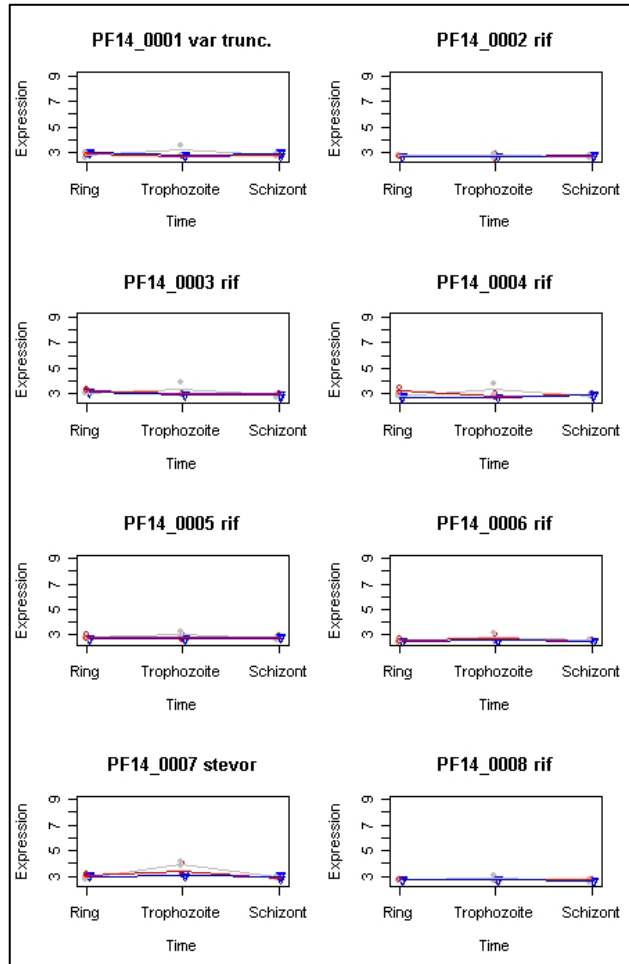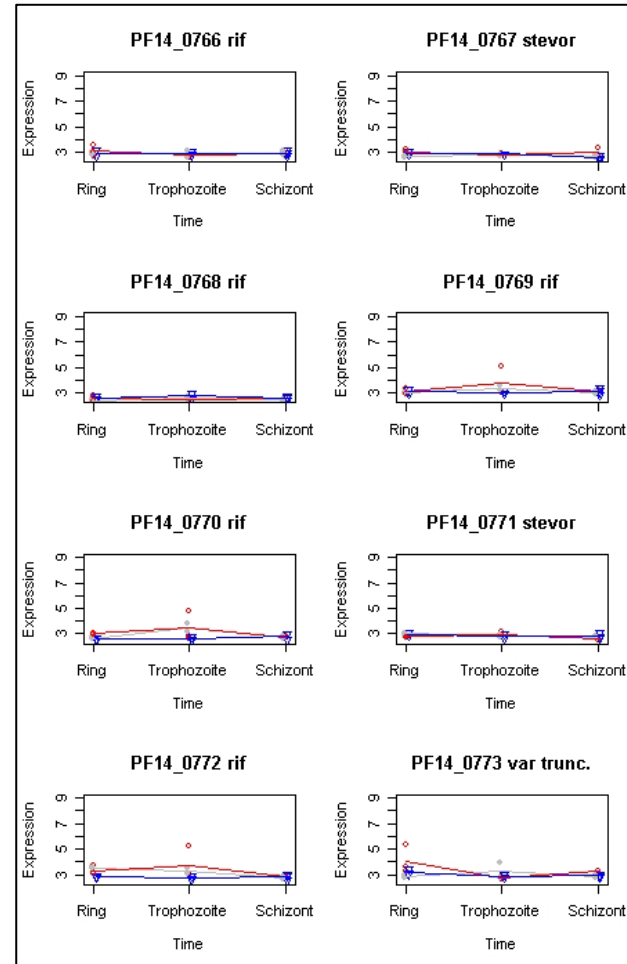

Supplement: Figure S3 [file pbio.1000084.sg003.pdf]

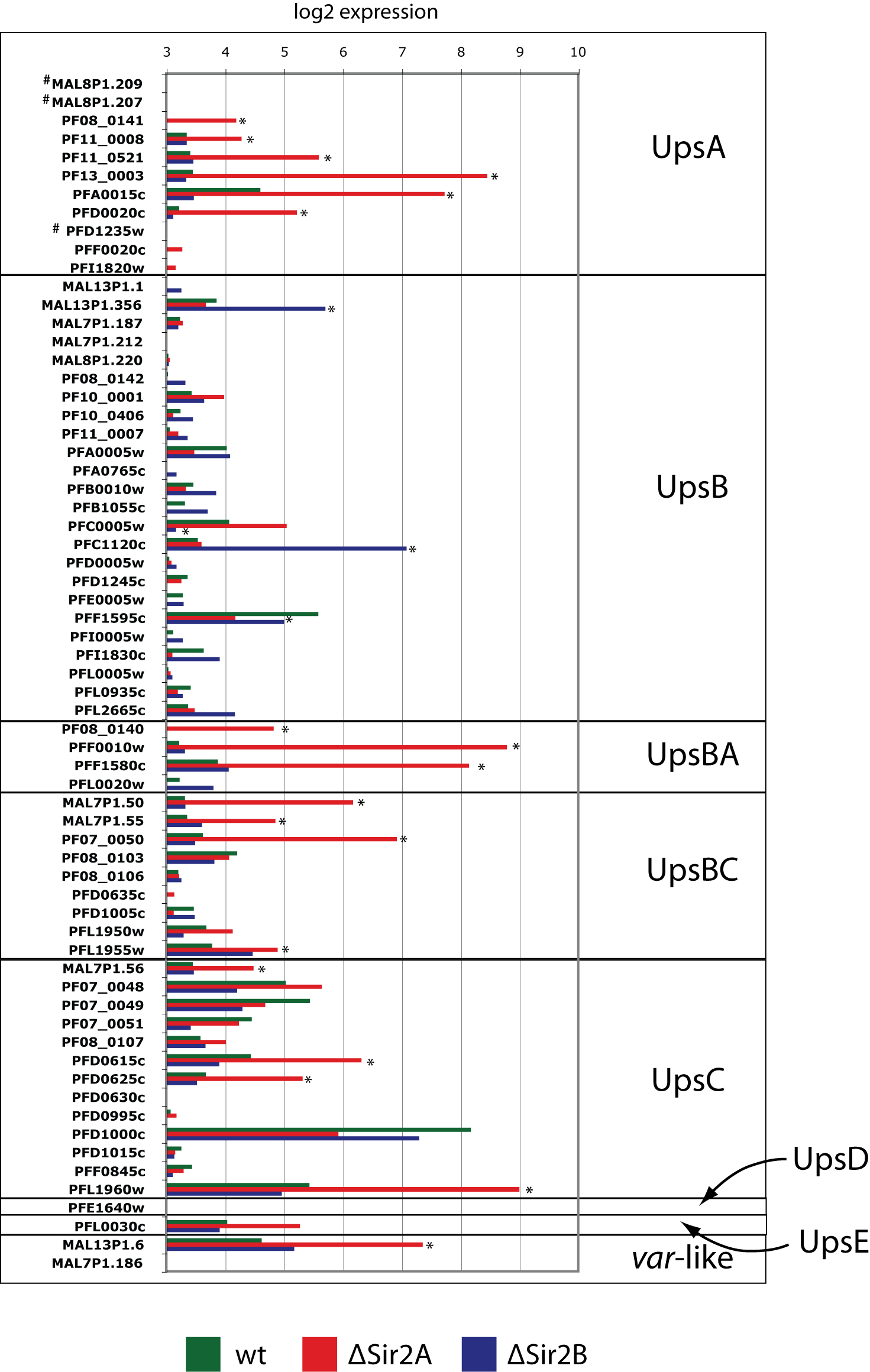

Supplement: Figure S4 [file pbio.1000084.sg004.tif]

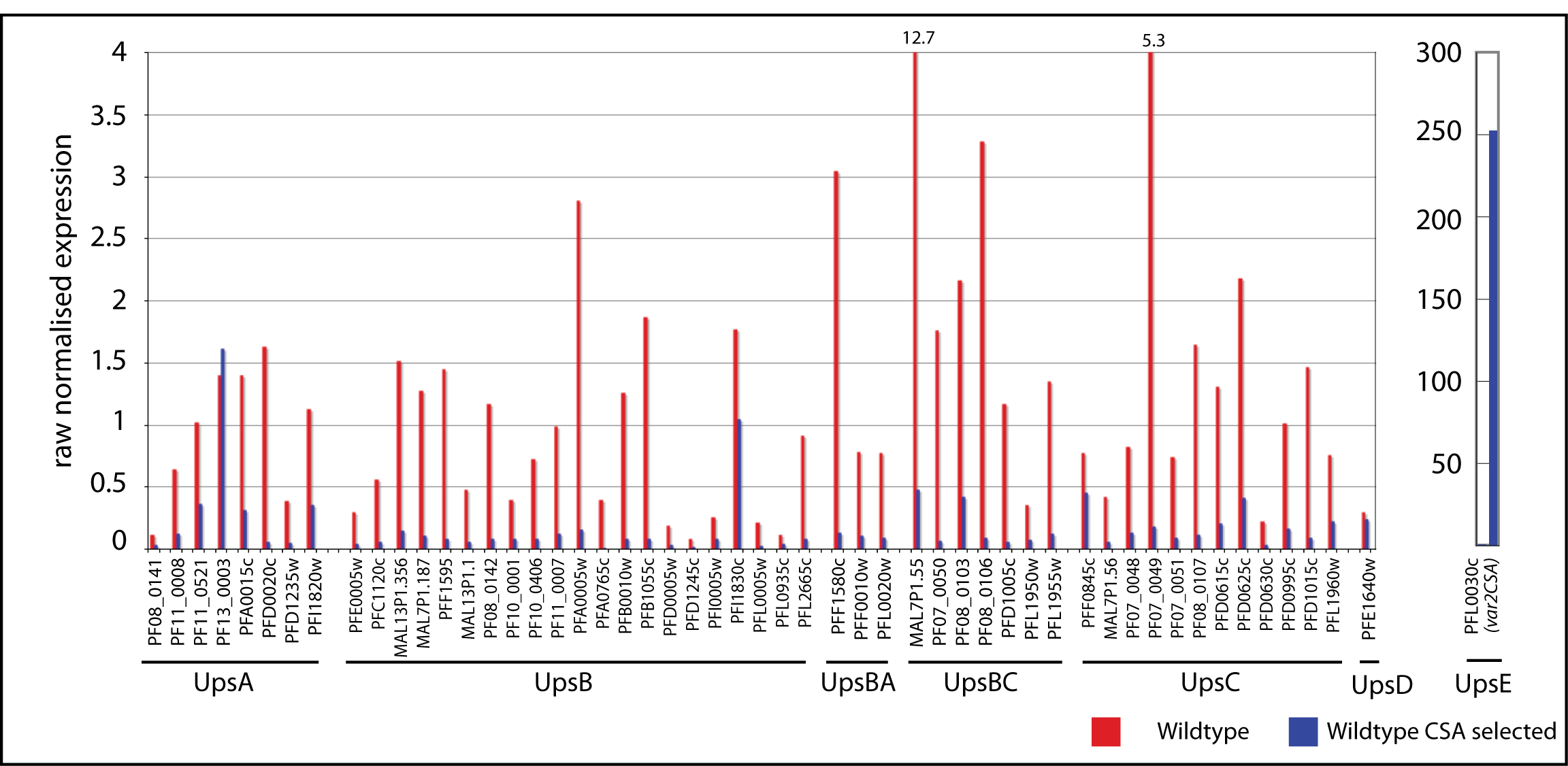

Supplement: Figure S5 [file pbio.1000084.sg005.tif]
