# Supplementary material for: Sir2 Paralogues Cooperate to Regulate Virulence Genes and Antigenic Variation in Plasmodium falciparum
Source: PLoS Biol. 2009 Apr 14;7(4):e1000084. doi: 10.1371/journal.pbio.1000084 (PMC2672602; doi:10.1371/journal.pbio.1000084)
Supplement: Text S1 — (52 KB DOC) [file pbio.1000084.sd001.doc]

Phylogenetic analysis of Sir2

We included in our phylogenetic analysis four sequenced Plasmodium species; Plasmodium vivax, Plasmodium chabaudi, Plasmodium yoelli, Plasmodium berghei, together with Sir2 homologues from other apicomplexan parasites; Toxoplasma gondii, Theileria annulata, Theileria parva and Cryptosporidium hominis. Within the analysis we also included Sir2 and Hst1 from Saccharomyces cerevisiae and the related Kluyveromyces lactis Sir2, which is considered the progenitor of the two S. cerevisiae Sir2 proteins [1]. All three yeast Sir2’s are considered type I sirtuins [2]. We also included three human sirtuins SIRT5 and SIRT6 and SIRT7. SIRT4 is a type III sirtuin, to which PfSir2A is also considered to belong, whilst SIRT6 and SIRT7 are group IV sirtuins [2]. Given that Apicomplexa are part of the larger chromalveolate phylogenetic group [3], we also included in the analysis three Sir2 homologues we found in a recently sequenced diatom species - Thalassiosira pseudonana. Last of all we also included in our analysis a Sir2 homologue from Giardia lamblia and Leishmania major – two other protozoan parasites.

Unrooted phylogenetic analysis of apicomplexan Sir2’s shows that the most paralogues fall into two distinct clades. Human SIRT6 and SIRT7 group with strong support (Red branches) with the Sir2B paralogues, further supporting the notion that the Sir2B in Apicomplexa are members of the group IV sirtuins (Fig. 1B). SIRT6 is involved in cellular aging, genomic stability and telomeric chromatin maintenance while SIRT7 is involved in the activation of RNA polymerase I. The functional consequence of SIRT6 and SIRT7 with apicomplexan Sir2B paralogues is not yet realised. Sir2A paralogues from all Plasmodium are highly similar (seen by the short branch lengths) and a Sir2 from T. gondii also groups with excellent support (blue branches; Fig. 1B). Previously, the P. falciparum Sir2A homologue was classified as a group III sirtuin to which human mitochondrial SIRT5 protein also belongs. SIRT5 groups with the Sir2A paralogues with moderate support (Fig. 1B). The type of tree building software used changed architecture of the SIRT4, L. major Sir2 (green branches) and the second T. pseudonana Sir2 paralogue suggesting that this is not a well-defined branch (data not shown). Interestingly, Theileria species only have one Sir2 and this groups strongly with Sir2B paralogues, and given the common topology of the apicomplexan tree it suggests that these species have lost their Sir2A paralogue (Fig. 1B). C. hominis is considered to be an early branching apicomplexan, it only has one Sir2 homologue and this does not fall into either the Sir2A or Sir2B clade under our analysis, but instead groups with the only G. lamblia homologue (light green branches). Genome sequence for the distantly related chromalveolate diatom is available [4] and we found three Sir2 paralogues within in this organism. Interestingly, one clearly groups with Sir2B/group IV sirtuins, the other with the C. hominis and G. lamblia Sir2, while the other is more like group II/III sirtuins (Fig. 1B). It is difficult to infer the ancestral state of Sir2 paralogues in this group of organisms given there seems to be multiple losses and duplication over evolutionary time.

Analysis of the catalytic domain of Sir2 paralogues

To unravel potential similarities and differences in function between Sir2 paralogues an alignment of the catalytic domain was created. P. falciparum and P. vivax Sir2B were aligned with Sir2A paralogues, one of which has been enzymatically characterised [5,6]. Other sirtuin family members including the S. cerevisiae sirtuin Hst2, which has been structurally solved, were also compared [7]. Our alignment shows that Sir2B, as do Sir2A paralogues have common features of structurally solved sirtuins [5,6]. The large NAD+ ‘Rossman fold’ binding pocket (marked with box’s 1, 2 and 3) is very well conserved (Fig. S1) and Plasmodium Sir2B paralogues also contain a cystine triad that binds zinc. There is however some diversity in the region that binds acetylated substrates suggesting a change in specificity between Plasmodium Sir2A and Sir2B paralogues (boxes 3 and 5; Fig. S1). Furthermore, the region that binds nicotinamide – a product of the deacetylation reaction which acts as an auto-inhibitibitory molecules contains a ~180 amino acid insertion, which is likely under low structural constraint considering the low complexity of primary sequence and the lack of conservation even between P. vivax and P. falciparum (Fig. S1). The N and C- terminus of Hst2 are involved in auto-regulation but Sir2B homologues are much larger and contain no similarity. No other regions within Sir2B molecules have similarity with any other proteins that might give a clue to their function.

Analysis of non-var, non-rifin differentially expressed genes in the Sir2-disrupted lines

We found that most differentially expressed genes between wildtype and the two PfSir2-disrupted lines are in ring-stage parasites, only a handful are found in trophozoite and schizont stages. We looked for common features of these genes, including; physical position along the chromosome, common predicted functions (by gene ontogeny (GO) annotation), presence of a signal peptide and PEXEL export element, a subtelomeric location (defined by 250 kb’s from telomere), time of maximal expression and number of transmembrane domains (Table S1).

We found that PfSir2A has the largest effect on gene expression in ring-stage parasites. Interestingly, most of these genes are down regulated in ΔPfSir2A suggesting that Sir2A somehow has a role in their activation. We could not find anything in common between any of these genes and GO analysis does not predict a common cellular function. A much smaller proportion of genes are differentially expressed in ring-stage parasites in the ΔPfSir2B and these too are down regulated compared to wildtype. Interestingly, these are mainly Plasmodium helical interdispersed subtelomeric family (PHIST) proteins. These all have signal peptides and PEXEL export elements and either a very early or very late intraerythrocytic transcriptional profile and a transmembrane domain. Previous bioinformatic analysis of the P. falciparum genome detects 72 members of the PHIST family, therefore those that are differentially expressed in the PfSir2-disrupted lines only represents a small proportion of these. Another PEXEL-containing gene family are differentially expressed in both Sir2-disrupted lines is PfMC-2TM members. This family consists of 12 members and closer analysis of this gene family shows that 10 out of 12 are differentially expressed in ΔSir2A. 1 of each of the stevor and etramp gene family members are affected by the removal of Sir2’s as well as one member of the acyl-CoA synthetase (PfACS) family. The list of all highly significantly differentially expressed genes can be found in the Supplementary table 1 (Excel spread sheet).

We also looked at other genes known to be involved in parasite phenotypic variation. The EBA and Rh molecules are involved in parasite invasion into the host erythrocyte. We found no differences in the expression of these molecules between wildtype and Sir2-disrupted lines.

In yeast rDNA genes are clustered together and are regulated by Sir2. Upon Sir2 removal yeast rDNA transcription increases, and furthermore, recombination and outlooping of repeats occurs and this latter process is associated with aging in yeast. Thus Sir2 is has been implicated in cellular aging. In P. falciparum rDNA genes are not found at one loci but are scattered throughout the genome and their expression level is not influenced by the removal of either PfSir2. P. falciparum can be grown in continuous culture and PfSir2-disrupted lines have never been seen to change in growth rate or any other pattern that could be associated with parasite aging.

References

1. Hickman MA, Rusche LN (2007) Substitution as a mechanism for genetic robustness: the duplicated deacetylases Hst1p and Sir2p in Saccharomyces cerevisiae. PLoS Genet 3: e126.

2. Frye RA (2000) Phylogenetic classification of prokaryotic and eukaryotic Sir2-like proteins. Biochem Biophys Res Commun 273: 793-798.

3. Fast NM, Kissinger JC, Roos DS, Keeling PJ (2001) Nuclear-encoded, plastid-targeted genes suggest a single common origin for apicomplexan and dinoflagellate plastids. Mol Biol Evol 18: 418-426.

4. Armbrust EV, Berges JA, Bowler C, Green BR, Martinez D, et al. (2004) The genome of the diatom Thalassiosira pseudonana: ecology, evolution, and metabolism. Science 306: 79-86.

5. Chakrabarty SP, Saikumari YK, Bopanna MP, Balaram H (2008) Biochemical characterization of Plasmodium falciparum Sir2, a NAD+-dependent deacetylase. Mol Biochem Parasitol 158: 139-151.

6. Merrick CJ, Duraisingh MT (2007) Plasmodium falciparum Sir2: an unusual sirtuin with dual histone deacetylase and ADP-ribosyltransferase activity. Eukaryot Cell 6: 2081-2091.

7. Zhao K, Chai X, Clements A, Marmorstein R (2003) Structure and autoregulation of the yeast Hst2 homolog of Sir2. Nat Struct Biol 10: 864-871.
